# P53-R273H mutation enhances colorectal cancer stemness through regulating specific lncRNAs

**DOI:** 10.1186/s13046-019-1375-9

**Published:** 2019-08-28

**Authors:** Yuechao Zhao, Yiran Li, Jie Sheng, Fan Wu, Kai Li, Rong Huang, Xiaojuan Wang, Tao Jiao, Xin Guan, Yan Lu, Xiao Chen, Zhiwen Luo, Yanchi Zhou, Hanjie Hu, Wenjie Liu, Boyu Du, Shiying Miao, Jianqiang Cai, Linfang Wang, Hong Zhao, Jianming Ying, Xinyu Bi, Wei Song

**Affiliations:** 1 Department of Biochemistry and Molecular Biology, State Key Laboratory of Medical Molecular Biology, Institute of Basic Medical Sciences Chinese Academy of Medical Sciences, School of Basic Medicine Peking Union Medical College, Beijing, 100005 China; 2 Department of Hepatobiliary Surgery, State Key Laboratory of Molecular Oncology, National Cancer Center/National Clinical Research Center for Cancer/Cancer Hospital, Chinese Academy of Medical Sciences and Peking Union Medical College, Beijing, 100021 China; 3 Department of Pathology, State Key Laboratory of Molecular Oncology, National Cancer Center/National Clinical Research Center for Cancer/Cancer Hospital, Chinese Academy of Medical Sciences and Peking Union Medical College, Beijing, 100021 China; 4 Department of Medical Biology, School of Basic Medical Sciences, Hubei University of Medicine, Shiyan, 442000 China

**Keywords:** p53, lncRNAs, Colorectal cancer, Cancer stem cell

## Abstract

**Background:**

TP53 is one of the most frequently mutated genes among all cancer types, and TP53 mutants occur more than 60% in colorectal cancer (CRC). Among all mutants, there are three hot spots, including p53-R175H, p53-R248W and p53-R273H. Emerging evidence attributes cancer carcinogenesis to cancer stem cells (CSCs). Long noncoding RNAs (lncRNAs) play crucial roles in maintaining the stemness of CSCs. However, it is unknown if mutant p53-regulated lncRNAs are implicated in the maintenance of CSC stemness.

**Methods:**

RNA-sequencing (RNA-seq) and ChIP-sequencing (ChIP-seq) were used to trace the lncRNA network regulated by p53-R273H in HCT116 endogenous p53 point mutant spheroid cells generated by the somatic cell knock-in method. RT-qPCR was used to detect lncRNA expression patterns, verifying the bioinformatics analysis. Transwell, spheroid formation, fluorescence activated cell sorter (FACS), xenograft nude mouse model, tumor frequency assessed by extreme limiting dilution analysis (ELDA), Western blot assays and chemoresistance analysis were performed to elucidate the functions and possible mechanism of lnc273–31 and lnc273–34 in cancer stem cells.

**Results:**

p53-R273H exhibited more characteristics of CSC than p53-R175H and p53-R248W. RNA-seq profiling identified 37 up regulated and 4 down regulated differentially expressed lncRNAs regulated by p53-R273H. Combined with ChIP-seq profiling, we further verified two lncRNAs, named as lnc273–31 and lnc273–34, were essential in the maintenance of CSC stemness. Further investigation illustrated that lnc273–31 or lnc273–34 depletion dramatically diminished colorectal cancer migration, invasion, cancer stem cell self-renewal and chemoresistance in vitro. Moreover, the absence of lnc273–31 or lnc273–34 dramatically delayed cancer initiation and tumorigenic cell frequency in vivo. Also, lnc273–31 and lnc273–34 have an impact on epithelial-to mesenchymal transition (EMT). Finally, lnc273–31 and lnc273–34 were significantly highly expressed in CRC tissues with p53-R273H mutation compared to those with wildtype p53.

**Conclusions:**

The present study unveiled a high-confidence set of lncRNAs regulated by p53-R273H specific in colorectal CSCs. Furthermore, we demonstrated that two of them, lnc273–31 and lnc273–34, were required for colorectal CSC self-renewal, tumor propagation and chemoresistance. Also, the expression of these two lncRNAs augmented in colorectal cancer patient samples with p53-R273H mutation. These two lncRNAs may serve as promising predictors for patients with p53-R273H mutation and are vital for chemotherapy.

**Electronic supplementary material:**

The online version of this article (10.1186/s13046-019-1375-9) contains supplementary material, which is available to authorized users.

## Background

Evidence has indicated that 90% of mortality from cancer is attributable to metastases [1], including colorectal cancer [2]. Cancer stem cells (CSCs) display more migratory capability than other tumor cells [3]. Despite a small population, CSCs are considered as tumor-initiating cells in colorectal cancer and many other types of cancer. CSCs exhibit self-renewal ability and can propagate diverse cells that constitute the tumor [4]. A single allele mutation in TP53 leads to both loss of tumor-suppressive functions (LOF) and gain of oncogenic functions (GOF) [5]. Among single allele mutant types, there are three ‘hot-spot’ mutations that are categorized into the following two types: conformational mutations, such as R175H; and DNA-contact mutations represented by R248W and R273H [6]. Recent studies have demonstrated that somatic mutations in TP53 (p53-R273H) play critical roles in chemotherapy-induced colorectal CSC, but the underlying lncRNAs changes invovled in this process is not completely understood [7].

Long noncoding RNAs (lncRNAs) have been reported to play critical roles in maintaining stemness of cancer stem cells and chemoresistance [8]. Abnormal expression of lncRNAs reflects their different roles in the progression of colorectal cancer [9, 10]. Increasing evidence has shown that many lncRNAs play roles in the pathogenesis and progression of different kinds of cancers [11]. It had been reported that wildtype p53, as an important transcript factor, could regulate lncRNAs transcription under DNA damage or UV stress [12, 13]. Thus, considering the robust transcript function of mutant p53 in cancer development [14, 15], it is reasonable to speculate that mutant p53 may change its downstream lncRNAs regulation networks and endow tumor cells with stemness. However, a comprehensive mutant p53-regulated lncRNA network in colorectal CSC remain elusive.

In this study, we integrate genome-wide expression data obtained by RNA sequencing (RNA-seq) with p53-R273H ChIP-seq data of human colorectal cancer cells in CSC state. The combination of these experimental approaches allowed us to illustrate p53-R273H specific regulated lncRNA network, and two of the lncRNAs were found to be required for colorectal CSC self-renewal, tumor propagation and chemoresistance. We also show that the expression of p53-R273H-regualed lncRNAs increased in colorectal cancer patient samples with p53-R273H mutation. In summary, we defined a high-confidence set of 41 lncRNAs that are p53-R273H transcriptional targets and demonstrated two of the p53-R273H-regulated lncRNAs are required for colorectal cancer stemness maintenance and chemoresistance.

## Methods

### Cell lines and cell culture

The human HCT116 CRC cell line was obtained from the Cell Resource Center of Peking Union Medical College (Beijing, China). The HCT116 p53−/− cell line was generated in our lab. HCT116 p53−/− and HCT116 p53 endogenous point mutant cell lines were cultured in Iscove’s Modified Dulbecco’s Medium (HyClone, USA) with 10% fetal bovine serum (FBS, Gibco, USA) and 1% penicillin/streptomycin (Gibco, USA). When cells reached 80–90% confluence, cell passage was conducted, and cells in logarithmic growth phase were collected for use. Cells were confirmed to be free of mycoplasma using the Bimake mycoplasma detection kit. HCT116 p53 endogenous point mutant cell lines were constructed as shown in Additional file 1, S2A.

### Cell migration and invasion assay

The cell invasive and migratory ability was detected using an 8 μm pored, 6.5 mm polycarbonate transwell filter (Corning, USA), according to our previous study [16]. For the cell migration assay, 5 × 10^5^ cells in serum-free IMDM were seeded in the upper chamber and the lower chamber contained complete IMDM with 10% FBS (0.5 mL). After incubation for 36–48 h, the cells were fixed with paraformaldehyde and stained with 0.1% Crystal Violet (Beyotime, Jiangsu, China) for 0.5 h. Cells were counted using a microscope. For the cell invasion assay, chamber was uniformly coated with Matrigel basement membrane matrix (BD Biosciences, USA) and incubated at 37 °C for 4 h to form the Matrigel layer in the chamber. The following procedures were the same as migration assay. The migration and invasion experiments were performed in triplicate.

### Spheroid-forming assay

The spheroid-forming assay was conducted as previous study with little modification [17]. Cells lines were plated in 6-well, ultra-low attachment plates (Corning Life Sciences) at a density of 3000 viable cells per well. Cells were grown in spheroid medium consisting of DMEM/F12 (Invitrogen) supplemented with B27 serum-free supplement (1:50; Invitrogen), 20 ng/ml epidermal growth factor (R&D Systems), 20 ng/ml basic fibroblast growth factor (R&D Systems), and penicillin-streptomycin at 37 °C in 5% CO_2_. The experiment was terminated after 10–12 days, and the spheroids were quantified.

### RNA isolation and reverse transcription quantitative PCR (RT-qPCR)

Total RNA derived from spheroids or cells was extracted using RNeasy Plus Mini Kit (QIAGEN) according to the manufacturer’s protocols. A total of 1 μg RNA sample was used to synthesized first-strand cDNA using random hexamers and RevertAid First Strand cDNA Synthesis Kit (Thermo Fisher Scientific) according to manufacturer’s instructions. Reverse transcription quantitative PCR (RT-qPCR) reactions including 1 μL cDNA were carried out in 20-μL reactions using PowerUp™ SYBR™ Green Master Mix (Applied Biosystems) and 0.5 mM specific primers performed by a BioRad CFX Manager Real-Time PCR system. The cycling conditions comprised 10 min polymerase activation at 95 °C and 40 cycles at 95 °C for 15 s and 60 °C for 1 min. Actin was used as an internal control. All samples were normalized to internal controls, and fold changes were calculated through relative quantification as follows: 2-[(Ct of gene)-(Ct of Actin)]. Primer sequences are listed in Additional file 1: Table S1.

### LncRNA interference and cell transfection

ASO pools targeting lnc273–31 and lnc273–34 as well as one negative control ASO pool were obtained from Ribo Tech (Shanghai, China). Briefly, 50–100 nM of each ASO pool was introduced in cells using RNAiMAX Transfection Reagent (Invitrogen) according to the manufacturer’s instructions. Sequences of ASO targeting lnc273–31 or lnc273–34 are shown in Additional file 1: Table S2. Corresponding negative control (NC) was carried out simultaneously.

### Induction of oxaliplatin resistant cell lines

The construction of chemo-resistant cell lines were constructed according to these two studies with a little modification [18, 19]. Oxaliplatin-resistant HCT116 p53 endogenous point mutant cell lines were derived from original parental cell line by continuous exposure to stepwise increasing concentrations of oxaliplatin (Bimake, USA). Initially, the exponentially growing cells were exposed to 2 μM oxaliplatin for 2–3 passages. The surviving cells were then exposed to gradually increasing concentrations of oxaliplatin from 0.5 μM to 1 μM during every passage. This development period was carried out for about 9 months. The final concentration of oxaliplatin is 20 μM. Generation of cell lines with acquired resistance to oxaliplatin was listed in Table 1. Additionally, vehicle treated parental cell line was kept in culture during this period as control cell line.

**Table 1:** Generation of cell lines with acquired resistance to oxaliplatin

Cell line	Initial Conc.	Final Conc.	Exposure passages	Exposure time
HCT116 p53-ctrl	2 μM	20 μM	53	9 months
HCT116 p53-R273H	2 μM	20 μM	53	9 months

### Cytotoxicity assay

Cytotoxicity assay was assessed using CCK8 (Cell Counting Kit-8, Dojindo, Japan) according to a previous study [20]. Cells were plated into 96-well plates at an initial density of 1 × 10^4^ cells/well for 24 h with 100 μL of medium and cultured with increased concentrations of oxaliplatin for 72 h. After the incubation, 10 μL of CCK-8 solution with 90 μL fresh medium was then added and incubated for 1 h. The absorbance was measured at 450 nm using an ELISA plate reader. Sensitivity to drugs was expressed in terms of the concentration of drug required to inhibit 50% of cell growth (IC50). The cell variability curves were plotted and IC50 values were determined through non-linear regression analysis using Graph Pad Prism software.

### Aldefluor assay and cell sorting

Cell populations with high ALDH1A1 enzymatic activity were identified with the Aldefluor kit (StemCell Technologies, Vancouver, BC, Canada) according to the manufacturer’s protocols. Briefly, 2 × 10^6^ cells were re-suspended in 1 mL Aldefluor buffer and 1 μL Aldefluor reagent in the presence or absence of the specific ALDH1A1 inhibitor for 30 min at 37 °C. Brightly fluorescent ALDH1A1-positive cells were detected in the green fluorescence channel, FL1, and samples treated with the specific ALDH1A1 inhibitor, DEBA, were used as the control to set the gates defining the ALDH1A1-positive region. Flow cytometry was performed using BD Accuri™ C6 instrument (BD Biosciences, San Jose, CA) as well as the data analyzing. For cell sorting, the cells were sorted a Moflo™ XDP high-performance cell sorter (Beckman Coulter, Brea, CA, USA). After sorting, the cells were washed and cultured for detection of lncRNA expression.

### Chromatin immunoprecipitation (ChIP)

ChIP assays were performed using a SimpleChIP Plus Sonication Chromatin IP Kit (Cell signaling Technology, #56383) with little modification. Spheroid cells (10^7^) were crosslinked with 1% formaldehyde for 5 min. Glycine was then added to a final concentration of 0.125 M for 5 min. Chromatin was disrupted by a Covaris S220 (power of 200 W and duty factor of 2.0) for 10 min. ChIP-qPCR primers are listed in Additional file 1: Table S3.

### Sequencing libraries preparation

Total RNA and ChIP DNA were isolated as described before. Novogene Technology Co., Ltd. (Tianjin, China) was trusted to prepare the libraries and perform the sequencing.

### RNA-sequencing data analysis

Paired-end and strand-specific RNA-seq libraries were prepared based on llumina instructions, and then sequenced on HiSeq 2000 (Illumina), of which the sequence length was set as 150 bp. GENCODE v27 (GRCh38.p10) assembly of the human genome was offered for annotation of the gene loci as reference. The HISAT2 [21] mapper (v2.1.0) generated the genome index and mapped reads to this human genome. StringTie [22] was applied to assemble the alignments, construct multiple isoforms and measure the expression of all genes and transcripts. Ballgown [23] was employed to analysis the expression levels of transcripts from StringTie and to estimate which transcripts were differentially expressed. LncRNAs or genes were considered significant if the false discovery rate (FDR) was less than 0.05.

### ChIP-sequencing data and p53 motif analysis

Bowtie (version 1.2.2) [24] was used to assemble raw sequence files to human genome. Peak detection and signal intensity analyses for ChIP-seq data were performed using MACS14 [25] and HOMER [26]. The binding site sequences matching to enriched regions were used to conduct the de novo motif search, which demanded the differentially selection of genes.

### Functional enrichment analysis

The Spearman rank correlation was devoted to detect the co-expression relationship between lncRNAs and protein-coding genes regulated by p53-R273H. A list of co-expressed genes of individual lncRNA regulated by p53-R273H were identified under a given threshold (coefficient > 0.9, coefficient < − 0.9 and FDR < 0.05). The potential combinative impact of lncRNA regulated by p53-R273H, was estimated by the functional enrichment analysis, containing the Gene Ontology (GO) and Kyoto Encyclopedia of Genes and Genomes (KEGG) pathways. A standard hypergeometric distribution was devoted to measure the significant *P*-values. Multiple hypothesis testing was performed using Benjamini & Hochberg (BH) method.

### Xenograft growth in nude mice

For subcutaneous injection models, serial dilutions (500, 5000, 50,000, 500,000 or 5000,000) of control and knock-down cells were implanted into two sides of the same nude mouse at the posterior dorsal flank region (male BALB/c nude mice; aged 4 to 6 weeks; *n* = 5 per group) with a Matrigel scaffold (BD Matrigel matrix, BD Biosciences). Tumors were measured every three days. Animal experiments were performed with the approval of Peking Union Medical College Animal Care and Use Committees. Mice were maintained under standard conditions according to the institutional guidelines for animal care. Tumorigenic cell frequency was calculated based on ELDA analysis (http://bioinf.wehi.edu.au/software/elda/). The statistical *p* value was obtained using a Chi-squared test.

### Tissue analyzes

The colorectal cancer FFPE tissues were obtained from the Cancer Hospital, Chinese Academy of Medical Sciences, used for RT-qPCR analysis. Prior patient consent and approval from the Institutional Research Ethics Committee were obtained for the use of these patient specimens for research purposes. The study was conformed to the ethical guidelines of the Declaration of Helsinki. The tissues were divided into two groups according to their p53 status detected by RNA-seq, which were p53 wildtype (*n* = 10) and p53-R273H mutation (*n* = 15). Total RNA derived from FFPE tissues was extrated using AllPrep DNA/RNA FFPE Kit (QIAGEN, 80234) according to manufacturer’ instruction.

### Statistical analysis

Data were presented as mean ± SD and analyzed by GraphPad Prism software. Student’s t-test was performed to analyze continuous variable. Association between lncRNA expression and clinicopathologic parameters were obtained from SPSS software. *P* < 0.05 was considered to be statistically significant.

## Results

### P53-R273H exhibits more invasive ability than p53-R175H and p53-R248W and promotes CSC expansion in vitro and in vivo

**Fig. 1:**
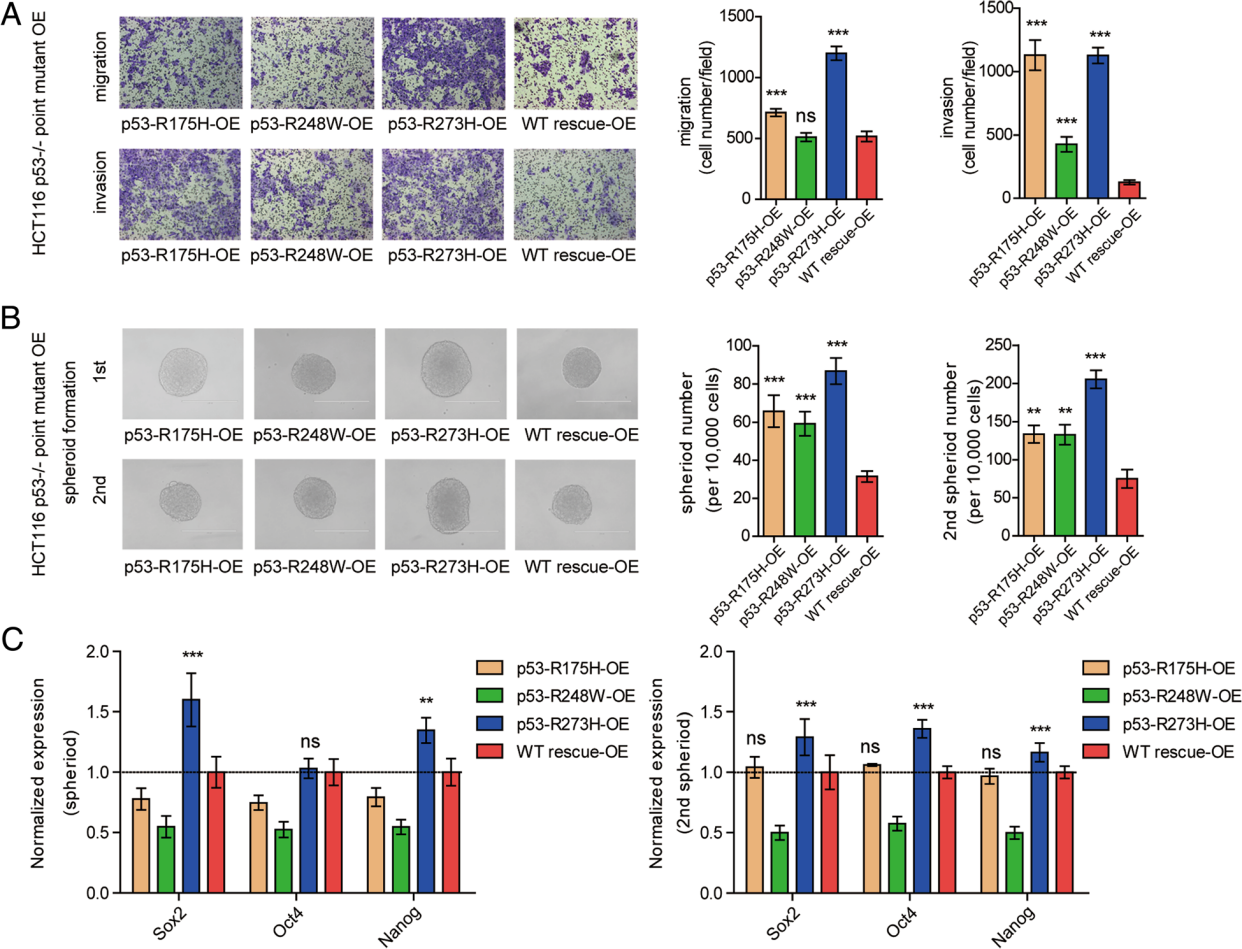
P53-R273H as to p53-R175H and p53-R248W exhibits more invasive ability and promote CSC expansion. **a**. Transwell assay of HCT116 p53−/− cells overexpression of R175H, R248W and R273H mutants. Representative images (left) and statistics (right) of migration and invasion assay were shown. OE, overexpression. **b**. The spheroids size (left) and number (right) of 1st spheroid formation (left, upper) and 2nd spheroid formation (left, down) assay of over-expression cell lines. Scale bar, 400 μM. **c**. Relative expression of stemness-related genes (Sox2, Oct4, Nanog) in over-expression cell lines was quantified by RT-qPCR. β-Actin mRNA was used as an internal control. The experiments were performed in triplicate. Data are shown as means ± SD. *p < 0.05, **p < 0.01, and ***p < 0.001

To examine the biological differences among the three hot-spot mutations in cancer stem cell state, R175H, R248W and R273H were overexpressed (OE) in HCT116 p53−/− cells and confirmed by Western blot (Additional file 1: Figure S1A). Migration assays showed that p53-R273H-OE had more significantly elevated migratory capabilities than p53-R175H-OE and p53-R248W-OE, which agreed with the invasion assay results (Fig. 1a). The roles of the three hot-spot mutations in CSC formation and self-renewal were next investigated. The spheroid-forming assay showed that all three mutations improved CSC formation, especially for p53-R273H-OE, as demonstrated by significantly increased spheroid size and numbers in first and secondary spheroid formation as compared to WT rescue-OE (Fig. 1b). The expression levels of cancer stemness-related genes, namely, Sox2, Oct4 and Nanog, were examined. Only the R273H mutation showed increased expression of these genes compared to WT rescue-OE (Fig. 1c). Collectively, these findings demonstrated that the p53-R273H mutation has more invasive and expansion ability compared to the other two p53 mutations.

**Fig. 2:**
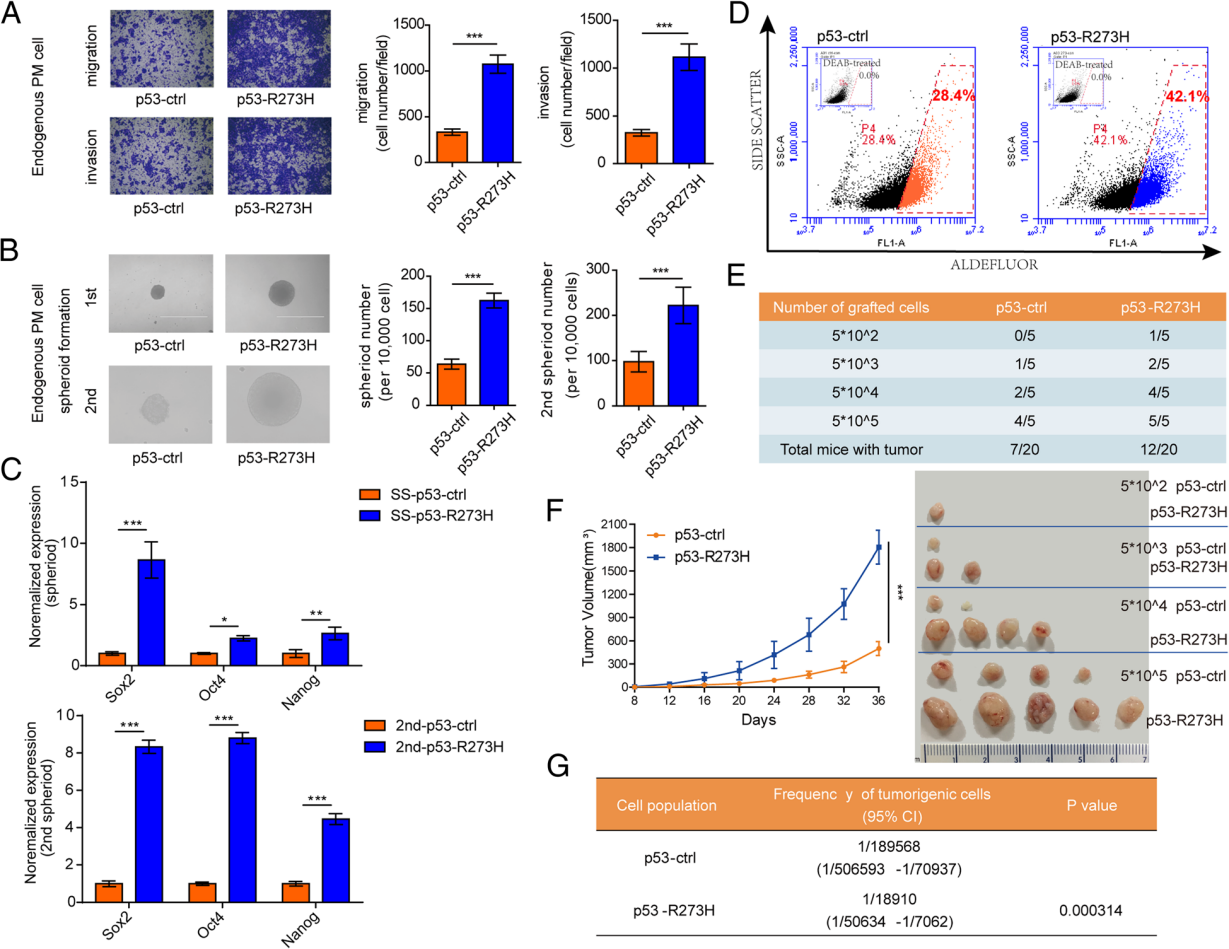
P53-R273H mutation promotes CSC expansion and in vitro and in vivo. **a**. Images (left) and statistics (right) of the migration assay and invasion assay of HCT116 p53 endogenous point mutant (PM) cell lines. **b**. The spheroids size (left) and number (right) of 1st spheroid formation and 2nd spheroid formation assay of HCT116 p53 endogenous point mutant (PM) cell lines. Scale bar, 400 μM. **c**. The relative expression of stemness-related genes (Sox2, Oct4, Nanog) in HCT116 p53 endogenous point mutant (PM) cell lines assessed by RT-qPCR analysis. **d**. ALDH1 activity was determined using the Aldefluor assay system in endogenous point mutant cells. ALDH1 inhibitor DEAB-treated cells were used as a negative control. Cell population with Aldefluor-derived fluorescence intensity was analyzed using flow cytometry. **e**. HCT116 p53 endogenous point mutant (PM) spheroid cells were diluted and subcutaneously implanted into BALB/c nude mice and tumor-free mice were monitored. *N* = 5 for each group. **f**. HCT116 p53 endogenous point mutant (PM) spheroid cells were subcutaneously injected into BALB/c nude mice to observe the growth of tumors. The tumor volumes were measured at the indicated time points (left), and images of tumors from nude mice at autopsy are presented (right). *N* = 5 for each group. G. Tumorigenic cell frequency of HCT116 p53 endogenous point mutant (PM) spheroid cells was determined with ELDA analysis (http://bioinf.wehi.edu.au/software/elda/). CI, confidence interval**.** The experiments were performed in triplicate (**a**-**d**). Data are shown as means ± SD. *p < 0.05, **p < 0.01, and ***p < 0.001

Endogenous heterozygous p53-R273H point mutant (p53-R273H-PM) HCT116 cells were generated using somatic cell knock-in technology as previously described (Additional file 1: Figure S2A) [27]. The cell model was confirmed by Sanger sequencing (Additional file 1: Figure S2B). As observed in p53-R273H-OE cells, p53-R273H-PM cells exhibited the similar increased invasive and migratory ability compared to p53-ctrl cells (Fig. 2a). Colorectal CSCs were enriched and self-renewal was assessed by 1st and 2nd spheroid formation. The spheroid size and numbers of p53-R273H mutation significantly increased when compared to p53-ctrl (Fig. 2b). And the expression of stemness-related genes was much higher in p53-R273H spheroids than that in p53-ctrl spheroids (Fig. 2c).

There are two widely recognized methods to measure the stemness of cancer cells, spheroid-forming assay and flow cytometry to analyze specific molecular markers. Next we further asked the percentage of CSC markers of colorectal caner stem cells, ALDH [28, 29]. The frequency of the ALDH^+^ population was determined using an Aldefluor assay. As shown in Fig. 2d, approximately 28.4% of the cells were ALDH^+^ in p53-ctrl cells while 42.1% in p53-R273H cells, which decreased to < 0.1% in both cells in the presence of diethylaminobenzaldehyde (DEAB) (an ALDH inhibitor), indicating the elevatory percentage of cancer stem cells in p53-R273H cells. In order to define the tumorigenic capacity of p53-R273H, p53-R273H-PM and p53-ctrl spheroid cells were subcutaneously implanted into BALB/c nude mice followed by an ELDA analysis. P53-R273H mutation resulted in a much stronger tumor presence compared with p53-ctrl spheroid cells as assessed by a limiting dilution xenograft analysis (Fig. 2e), suggesting that p53-R273H mutation increased tumor initiating capacity. P53-R273H led to 12 mice harboring tumors among 20 mice, while only 7 mice implanted with p53-ctrl harbored tumors (Fig. 2e). Moreover, p53-R273H mutation significantly increased xenograft tumor growth (Fig. 2f). Compared to p53-ctrl cells, p53-R273H-PM cells formed a tumor at a lower dilution rate (Fig. 2g), indicating the strong CSC frequency of p53-R273H. Overall, p53-R273H mutation enhances the tumorigenic capacity of colorectal CSCs.

### Global profiling and identification of p53-R273H-regulated lncRNAs

**Fig. 3:**
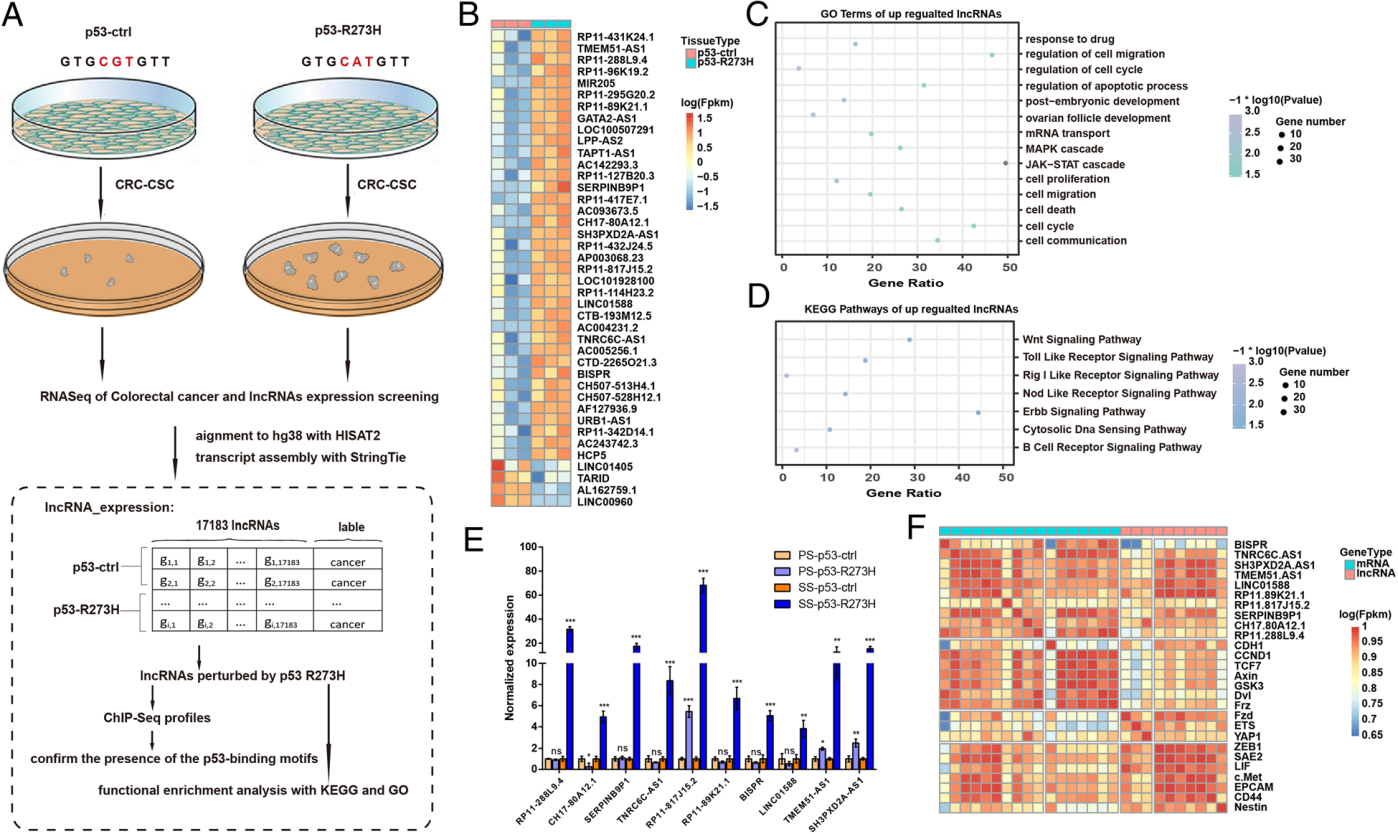
Global profiling and identification of p53-R273H-regulated lncRNAs. **a**. Schematic illustration of the experimental approach and the bioinformatics analysis workflow of this study. **b**. Gene expression profiles of HCT116 p53-R273H PM spheroid cells or p53-ctrl spheroid cells. Genes (fold change ≥2 or ≤ 0.5, *P* < 0.05) are shaded in blue or orange in the heat map to indicate p53-R273H groups or p53-ctrl groups, respectively. **c**. Functional annotation clustering of 41 differentially expressed lncRNAs according to GO analysis. **d**. Signaling pathway based on KEGG enrichment analysis of 41 differentially expressed lncRNAs. **e**. The relative expression level of the top 10 lncRNAs in plastic state (PS) cells and spheroid state (SS) cells determined by RT-qPCR. Data are shown as means ± SD. *p < 0.05, **p < 0.01, and ***p < 0.001. **f**. A heatmap of top 10 lncRNAs and CSC related genes. Red and blue represent correlation value greater than 0.95 or smaller than 0.8, respectively. R statistical software was used for visualization of the results

To unveil the genome-wide transcripts regulated by p53-R273H in the CSC state, HCT116 p53-R273H-PM spheroids were enriched, and the polyadenylated RNA fraction was isolated and used to perform strand-specific paired-end RNA Illumina sequencing. The workflow of the study design is illustrated in Fig. 3a. In total, 218,713 transcripts were successfully assembled, and 85% of the transcripts were annotated according to Gencode (version 27; GRCh38.p10) (Additional file 1: Figure S3A). Among the annotated transcripts, 75.6% (160,142) were identified as protein-coding mRNAs, and the remaining 24.4% were classified as different types of noncoding transcripts (Additional file 1: Figure S3B). In total, 13.4% (29,201) of transcripts were defined as lncRNAs, including antisense, intergenic, processed transcripts, sense-intronic and sense overlapping lncRNAs, and 2.3% (5032) of annotated transcripts corresponded to other types of noncoding RNAs, such as transcripts derived from pseudogenes, retained introns and pri-microRNAs (Additional file 1: Figure S3B).

To identify differentially expressed lncRNAs in cancer stem cells, p53-ctrl and p53-R273H spheroid cells were compared via Ballgown. In total, 41 lncRNAs were found differentially expressed with 37 up regulated and 4 down regulated (Fig. 3b; Additional file 2: adjust *P* < 0.05, log_2_Fold Change > 1 or < − 1). The validation results of these 41 lncRNAs were shown in Additional file 1: Figure S4A, using RT-qPCR. In summary, we have successfully validated 36 out of 37 up regulated lncRNAs and 3 out of 4 down regulated lncRNAs. Gene ontology (GO) analysis and KEGG pathway analysis were conducted on these up regulated lncRNAs to enrich the involved signaling pathways (Fig. 3c-d). CSC-related signaling pathways, such as drug resistance [30], JAK-STAT [31], MAPK [32] and Wnt [33] were enriched, further supporting the hypothesis that the subset of lncRNAs regulated by p53-R273H is specific to CSC. The functional analysis results of down regulated lncRNAs are listed in Additional file 3 and the differentially expressed protein coding genes are listed in Additional file 4.

The 36 up regulated lncRNAs were ranked by stemness score according to a previous description [34], and the top 10 lncRNAs were screened for further analysis, which namely as RP11 − 288 L9.4, CH17 − 80A12.1, SERPINB9P1, TNRC6C − AS1, RP11 − 817 J15.2, RP11 − 89 K21.1, BISPR, LINC01588, TMEM51 − AS1 and SH3PXD2A − AS1 (ranked 1 to 10) (Fig. 3e). To evaluate the co-expression relationship between the top 10 lncRNAs and well-known CSC-related genes, a heatmap of co-occurrence was generated with red and blue representing correlation values greater than 0.99 and less than 0.85, respectively. The top 10 lncRNAs had strong co-occurrence with the CSC-related genes (Fig. 3f), suggesting that lncRNAs regulated by p53-R273H are correlated with CSCs.

### Lnc273–31 and lnc273–34 are specifically regulated by p53-R273H in colorectal CSCs

**Fig. 4:**
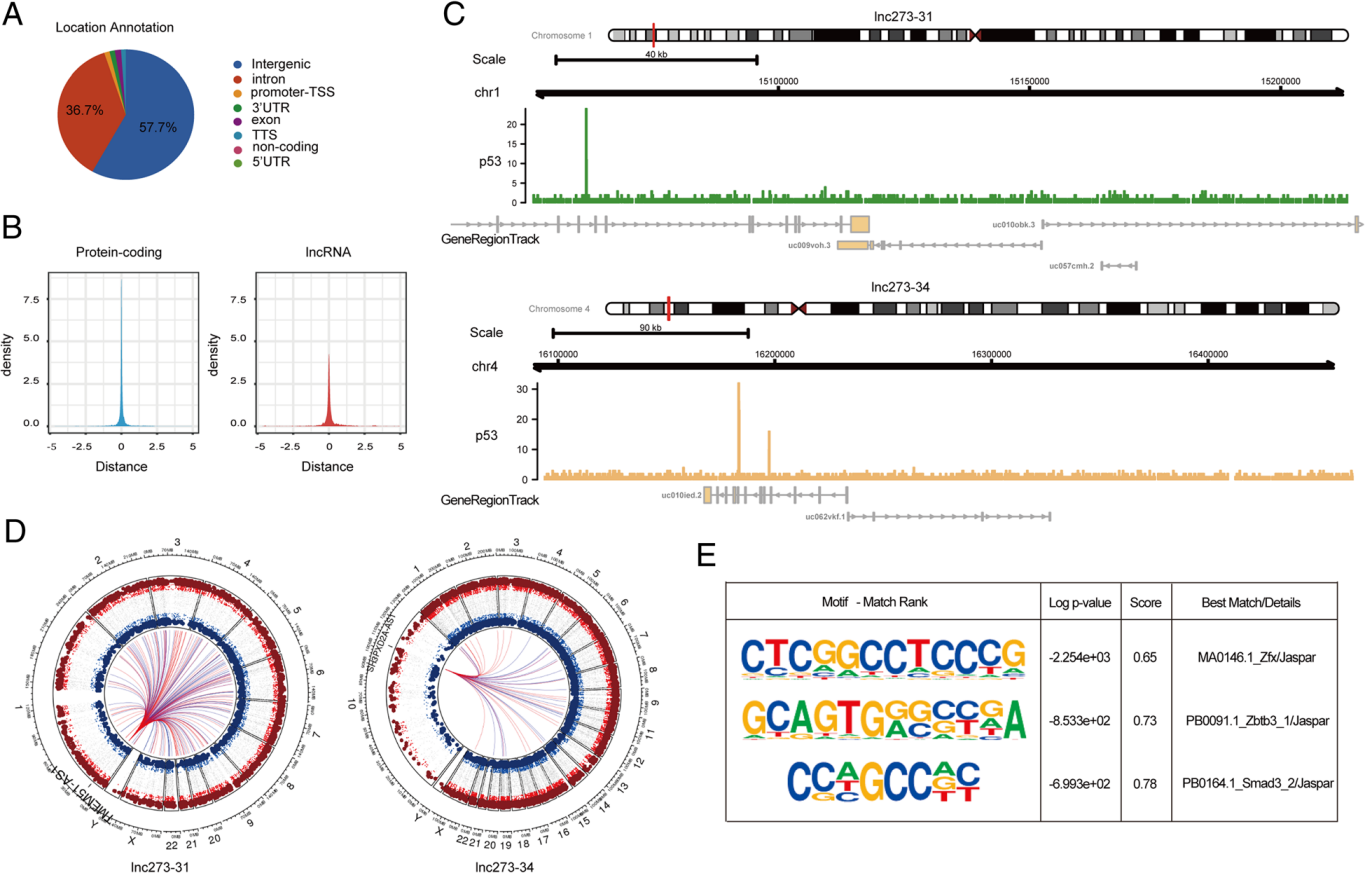
Lnc273–31 and lnc273–34 are specifically regulated by p53-R273H in colorectal CSCs. **a**. Pie chart illustrated the distribution of peak locations (promoter-TSS, TSS, intergenic, exon, intron, 5’UTR, 3’UTR and non-coding) in the genome. The pie chart shows the percentage of peaks in different genomic location. **b**. The overlap between significant peaks and transcription start sites (TSSs) for various gene classes (mRNAs and lncRNAs) are shown in left (blue) and right (red) of the density plot, respectively. **c**. Signals of p53 for lnc273–31 (green) and lnc273–34 (yellow), respectively. **d**. A Circos plot of the top 100 co-expressed genes related to the location of lnc273–31 (left) and lnc273–34 (right), respectively. **e**. HOMER was utilized to identify motifs. The range of nucleotides around the center of peaks for motif identification was set ±100 bp for establishing the primary and co-enriched motif bound by p53-R273H. Motif identification results were listed in a table including rank, log *p* value, score, best match/details. Enrichment *p*-values reported by HOMER was significant (< 0.05)

Considering the up regulated lncRNAs in p53-R273H cells may be caused by its GOF as a transcript factor, ChIP-seq was performed to discern p53-R273H direct targets. In the p53-R273H spheroid cells, 14,063 p53 peaks were identified, and 11,010 peaks were identified in p53-ctrl spheroid cells (Fig. 4a). Interestingly, the signal distribution of p53 peaks bound with mRNAs was within ten, while peaks associated with lncRNAs were less than five (Fig. 4b). In total, 3840 lncRNAs were regulated by p53-R273H, and 1196 of which were annotated by Gencode v27 as intergenic lncRNAs (608), antisense lncRNAs (566) or processed transcripts (22) (Additional file 5).

To further trace p53-R273H regulated lncRNAs in the CSC state, ChIP-seq analysis was combined with RNA-seq analysis, which identified 7 lncRNAs regulated by p53-R273H (short for lnc273s, Additional file 6). Among these lncRNAs, two lncRNAs, which were also present in the top 10 lncRNAs ranked by stemness score, and named TMEM51-AS1 as lnc273–31 and SH3PXD2A-AS1 as lnc273–34 (Fig. 4c). A circos plot was then generated to show the genome-wide distribution of the top 100 co-expressed genes related to the location of lnc273–31 and lnc273–34 using circlncRNAnet [35] (Fig. 4d). Consistent with the binding of p53, the p53 consensus motif was highly enriched across the p53-bound loci (Fig. 4e), confirming the presence of p53REs in these genomic regions.

### Knockdown of lnc273–31 or lnc273–34 inhibits the self-renewal maintenance of colorectal CSC in vitro and in vivo

**Fig. 5:**
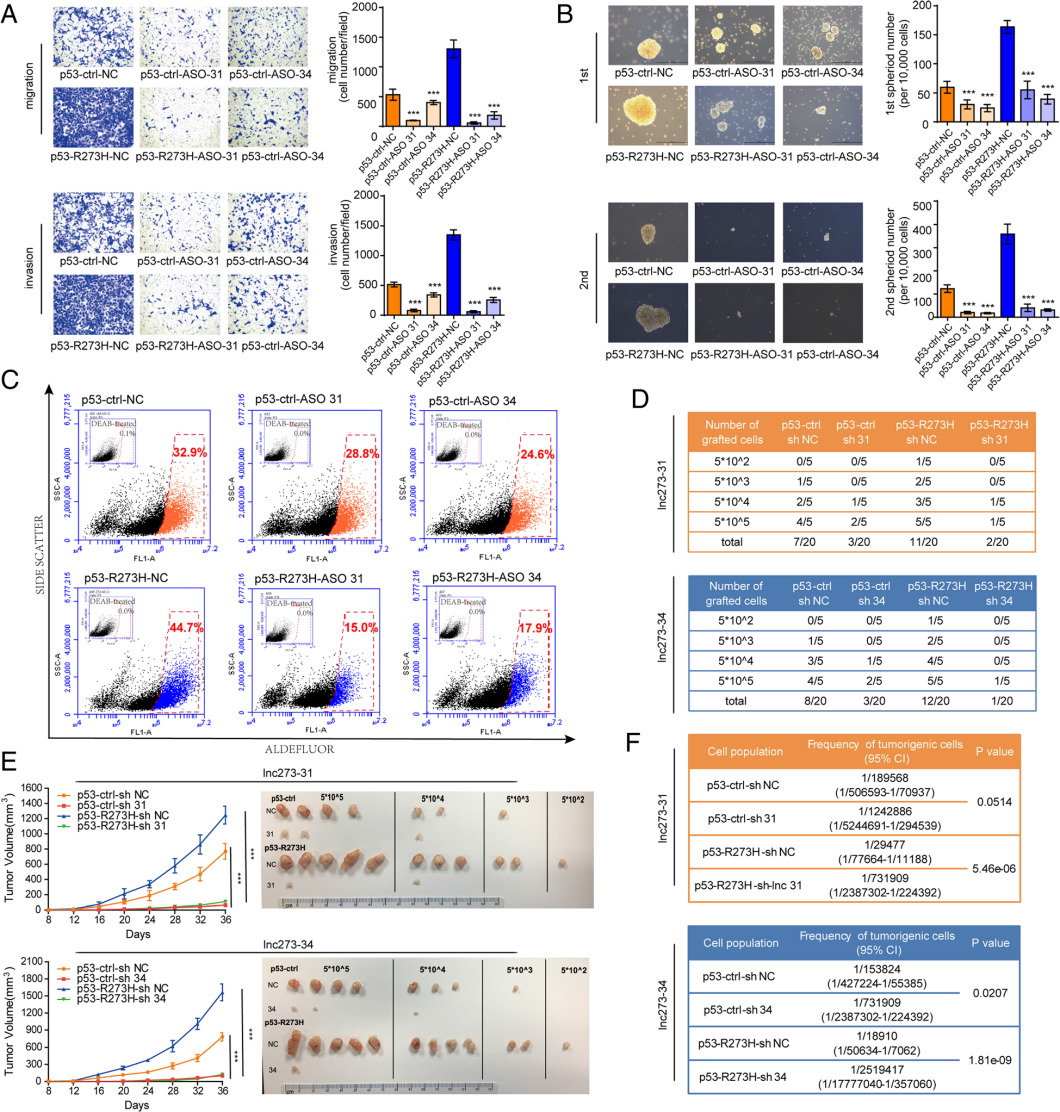
Knockdown of lnc273–31 or lnc273–34 inhibits the self-renewal maintenance of colorectal CSC in vitro and in vivo. **a**. Migratory and invasive ability of HCT116 p53 endogenous point mutant (PM) cells with lnc273–31 (upper) or lnc273–34 (down) knockdown were measured by transwell migration and matrigel invasion assays using ASOs. ASO, antisense oligos. **b**. The spheroids size (left) and numbers (right) of 1st spheroid formation (upper) and 2nd spheroid formation (down) assay after lnc273–31 (upper) or lnc273–34 (down) silenced in HCT116 p53 endogenous point mutant (PM) cells. Scale bar, 500 μM. **c**. ALDH enzymatic activity was assessed using the Aldefluor assay system in lnc273–31 or lnc273–34 silencing cells. **d**. Stable lnc273–31/34-knockdown or shNC spheroid cells were diluted and subcutaneously implanted into BALB/c nude mice and tumor-free mice were monitored. *N* = 5 for each group. **e**. Stable lnc273–31/34-knockdown or shNC spheroid cells were subcutaneously injected into BALB/c nude mice for observation of tumor growth. The tumor volumes were measured at the indicated time points (left), and images of tumors from nude mice at autopsy are presented (right). **f**. Tumorigenic cell frequency in stable lnc273–31/34-knockdown or shNC spheroid cells was determined with ELDA analysis (http://bioinf.wehi.edu.au/software/elda/). CI, confidence interval. The experiments were performed in triplicate (**a**-**c**). Data are shown as means ± SD. *p < 0.05, **p < 0.01, and ***p < 0.001

To determine the functions of lnc273–31 and lnc273–34 in colorectal cancer cells, antisense oligonucleotides (ASOs) were designed to reduce target lncRNA expression levels. Knock-down of lnc273–31 or lnc273–34 in either p53-ctrl cells or p53-R273H cells, dramatically decreased the invasive and migratory capability compared to negative control (NC), especially in p53-R273H cells (Fig. 5a). Furthermore, knocking down of lnc273–31 or lnc273–34 significantly impaired the spheroid formation abilities, including spheroid size and spheroid number, indicating the attenuated generation of colorectal CSCs and self-renewal capabilities (Fig. 5b). Moreover, lnc273–31 and lnc273–34 depletion significantly reduced the expression of stemness-related genes compared to NC, shown in Additional file 1: Figure S7A.

Next we detected the frequency of the ALDH^+^ population in lnc273–31 or lnc273–34 depleted cells. In p53-ctrl cells, the frequency of the ALDH^+^ population decreased from 32.9% in NC cells to 28.8% or 24.6%, when knocking down lnc273–31 or lnc273–34, respectively. While in p53-R273H cells, ALDH positive percentage decreased from 44.7% in NC cells to 15.0% or 17.9%, when lnc273–31 or lnc273–34 depletion, respectively (Fig. 5c). This results further supported the conclusion that p53-R273H-regulated lncRNAs are essential for the CSC maintenance.

The effect of lnc273–31 and lnc273–34 on tumor-initiating capacity was next assessed by ELDA analysis. The p53-R273H and p53-ctrl spheroid cells with stable lnc273–31 or lnc273–34 knockdown as well as the respective NC spheroid cells were subcutaneously injected into BALB/c nude mice. Lnc273–31 or lnc273–34 depletion resulted in more tumor-free mice compared to NC, indicating lnc273–31 and lnc273–34 depletion attenuated tumor initiating capacity. Two or one out of 20 mice harbored tumors with lnc273–31 or lnc273–34 knockdown, respectively, while 11 or 12 mice harbored tumors in the NC group (Fig. 5d). The results were more significant in p53-R273H groups compared to p53-ctrl (3 out of 20 mice harbored tumors in p53-ctrl groups compared to 1 or 2 mice in knockdown groups), suggesting that lnc273–31 and lnc273–34 are closely related to tumor-initiating capacity. Meanwhile, knockdown of lnc273–31 or lnc273–34 significantly reduced the size of xenograft tumor growth (Fig. 5e). The tumorigenic cell frequency decreased sharply after knockdown of lnc273–31 or lnc273–34 (Fig. 5f). Overall, lnc273–31 or lnc273–34 silencing abrogates the tumorigenic capacity of colorectal CSCs.

### P53-R273H-regulated lncRNAs are implicated in oxaliplatin resistance in colorectal cancer stem cells

**Fig. 6:**
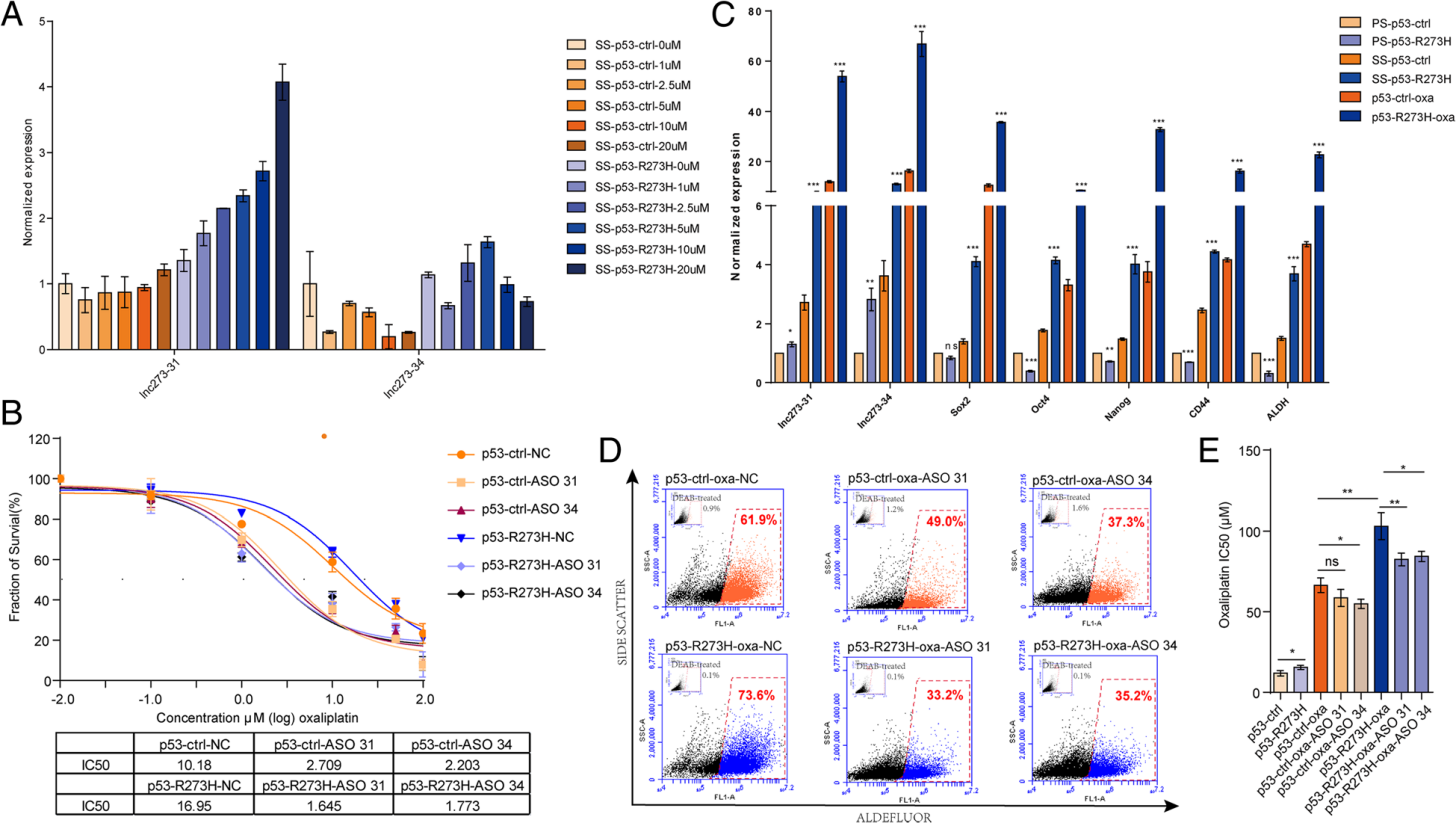
P53-R273H-regulated lncRNAs are implicated in oxaliplatin resistance in colorectal cancer stem cells. **a**. HCT116 p53 endogenous point mutant (PM) spheroid cells were treated with oxaliplatin (0, 1, 2.5, 5, 10, 20 μM) for 24 h. The relative expression of lnc273–31 and lnc273–34 was assessed by RT-qPCR. **b**. The IC50 changes after knocking down of lnc273–31 or lnc273–34 in HCT116 p53 endogenous point mutant cells. **c**. The relative expression of stemness related genes (Sox2, Oct4, Nanog, CD44, ALDH) in HCT116 p53 endogenous point mutant plastic cells (PS), spheroid cells (SS) and oxaliplatin resistant cells (oxa). **d**. Oxaliplatin-resistant cells in the presence of lnc273–31 or lnc273–34 knock-down were stained with the Aldefluor assay to determine the frequencies of ALDH^+^ cells vs non-treated cells. Experiments were normalized with respective DEAB controls. **e**. The IC50 changes of knocking down of lnc273–31 or lnc273–34 in oxaliplatin resistant cells. The experiments were performed in triplicate (**a**-**e**). Data are shown as means ± SD. *p < 0.05, **p < 0.01, and ***p < 0.001

To determine if lnc273–31 and lnc273–34 react with oxaliplatin, p53-R273H spheroids were exposed to different concentrations of oxaliplatin, and the expression of lncRNAs was examined. Surprisingly, oxaliplatin treatment induced lnc273–31 expression in a concentration-dependent manner, but the expression of lnc273–34 did not show the same trend (Fig. 6a). Further analysis of the expression levels of lnc273–34 in p53-ctrl and p53-R273H cells demonstated that the expression level in p53-R273H cells was much higher than that in p53-ctrl cells, indicating the regulatory function of p53-R273H in this chemo-reaction process.

Knockdown of either lnc273–31 or lnc273–34 significantly decreased the IC50 of oxaliplatin in p53-ctrl cells from 10.18 to 2.709 or 2.203, and the decrease in the IC50 of oxaliplatin in p53-R273H was more significant from 16.95 to 1.645 or 1.773 (Fig. 6b). Next we constructed endogenous p53 PM cell lines resistant to oxaliplatin, which were named as p53-ctrl-oxa and p53-R273H-oxa. In oxaliplatin-resistant cell lines, the expression levels of lnc273–31, lnc273–34 and stemness-related genes were much higher than those in the parental cell lines with more than 10-fold increases in p53-R273H-oxa and at least 3-fold increases in p53-ctrl-oxa cells (Fig. 6c). The expression levels were also much higher in oxaliplatin-resistant cell lines than those in spheroid cells with 4- and 11-fold increases, and the expression levels were higher in p53-R273H-oxa cells than in p53-ctrl-oxa cells (Fig. 6c).

We next found that the frequency of the ALDH^+^ population in oxaliplatin-resistant cell lines was higher than their parental cell lines (approximately 28.4% of the cells were ALDH^+^ in p53-ctrl cells and 42.1% in p53-R273H cells, while 61.9% in p53-ctrl-oxa cells and 73.6% in p53-R273H-oxa cells, Fig. 2d, 6d), indicating the oxaliplatin induction of CSCs. When knocking down of lnc273–31 or lnc273–34, the ALDH positive percentage in p53-ctrl-oxa cells dropped from 61.9% in NC cells to 49.0% or 37.3%, respectively. While in p53-R273H cells, the frequency of the ALDH^+^ population decreased from 73.6% in NC cells to 33.2% or 35.2% (Fig. 6d). Furthermore, knockdown of lnc273–31 or lnc273–34 in oxaliplatin-resistant cell lines decreased the IC50 of oxaliplatin more significantly in p53-R273H-oxa than in p53-ctrl-oxa (Fig. 6e). In summary, lnc273–31 and lnc273–34 are implicated in oxaliplatin resistance in colorectal cancer.

### The expression of lnc273–31 and lnc273–34 was significantly up regulated in CRC tissues with p53-R273H mutation as compared to p53 wildtype tissues

**Fig. 7:**
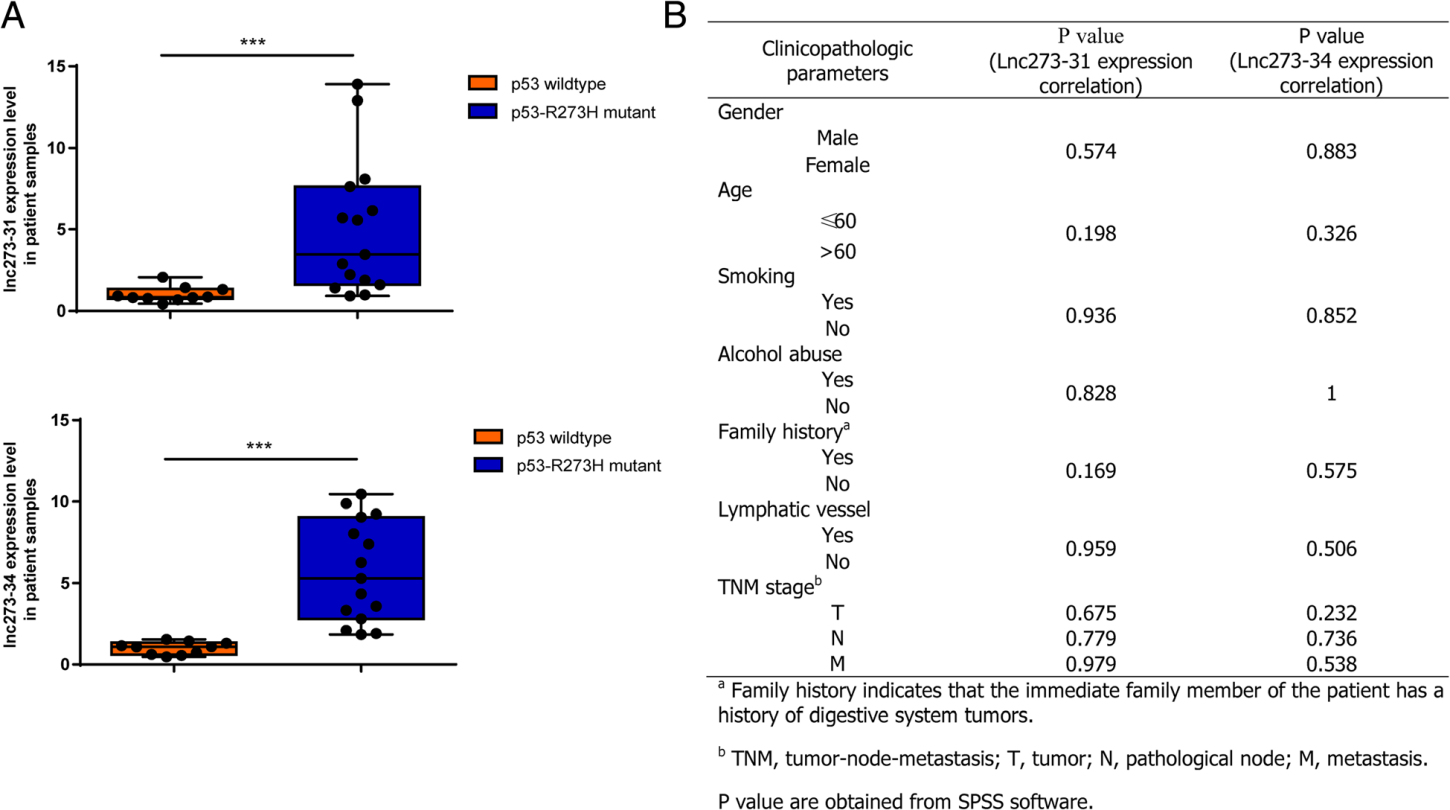
The expression of lnc273–31 and lnc273–34 was significantly up regulated in CRC tissues with p53-R273H mutation as compared to p53 wildtype tissues. **a**. RT-qPCR analysis of relative lcn273–31 and lnc273–34 expression in colorectal cancer FFPE tissue samples. The expression of lncRNAs was normalized to that of β-actin. Data are shown as means ± SD. **b**. Association between lncRNA expression and clinicopathologic parameters in 25 colorectal cancer patients with or without p53-R273H mutation

To explore the relationship between lnc273–31, lnc273–34 and p53-R273H mutation in colorectal cancer patients, we detected the expression level of lnc273–31 and lnc273–34 using RT-qPCR method in colorectal cancer tissue samples with or without p53-R273H mutation. The patients were divided into two groups according to their p53 status (Additional file 7). The expression level of lnc273–31 and lnc273–34 in colorectal cancer patients with p53-R273H mutation is higher than that in p53 wildtype group (Fig. 7a), and the difference was statistically significant (*p* < 0.001). Also we further analyze the association of age, gender, smoking, alcohol abuse, family history, lymphatic vessel, TNM stage and the levels of lncRNAs, and we found that lncRNA expression was nonrelevant with these clinicopathologic parameters (Fig. 7b, Additional file 1: Figure S8). All together, this finding further supported the previous results in our study, indicating that dysregulation of lnc273–31 or lnc273–34 expression have strong relevance with p53-R273H, leading to priming of the self-renewal of colorectal CSCs and tumor initiation.

## Discussion

Cancer stem cells have been considered as the core origin of the tumorigenesis and therapeutic resistance. For this reason, they have also been termed “tumor-initiating cells” [36]. As non-coding RNAs being found overwhelming majority of the transcripts, more and more studies have illustrated their roles in cancer development, as well as in cancer stem cells [37]. Given the robust transcript capability of mutant p53, we aimed to identify lncRNAs regulated by mutant p53, endowing cells with stem-like properties. The present study unveiled a subset of lncRNAs regulated by p53-R273H to be essential in cancer stemness maintenance and chemoresistance, through RNA-seq combined with ChIP-seq.

Mutant p53 have been found to gain of function in tumor malignancy, and different types of mutation have diverse functions in cancer development, which further explains the gain-of-function of mutant p53. Previous studies on p53-R175H or p53-R248W have mainly focused on their effects on tumor propagation [38, 39]. P53-R273H has been shown to induce drug resistance through downregulating procaspase-3 levels [40] and promoting chronic inflammation and inflammation-associated cancer [41]. Most of these studies were based on mutant p53, as an oncogenic gene. Few studies have been reported on mutant p53 as an oncogenic transcription factor, to expore its function of regulating noncoding RNAs. Although there are several studies about mutant p53 affecting well-studied lncRNAs [42], in different cancer types, a systematic analysis of specific lncRNA networks regulated by p53-R273H in the CSC state was lacking until the present study was performed.

**Fig. 8:**
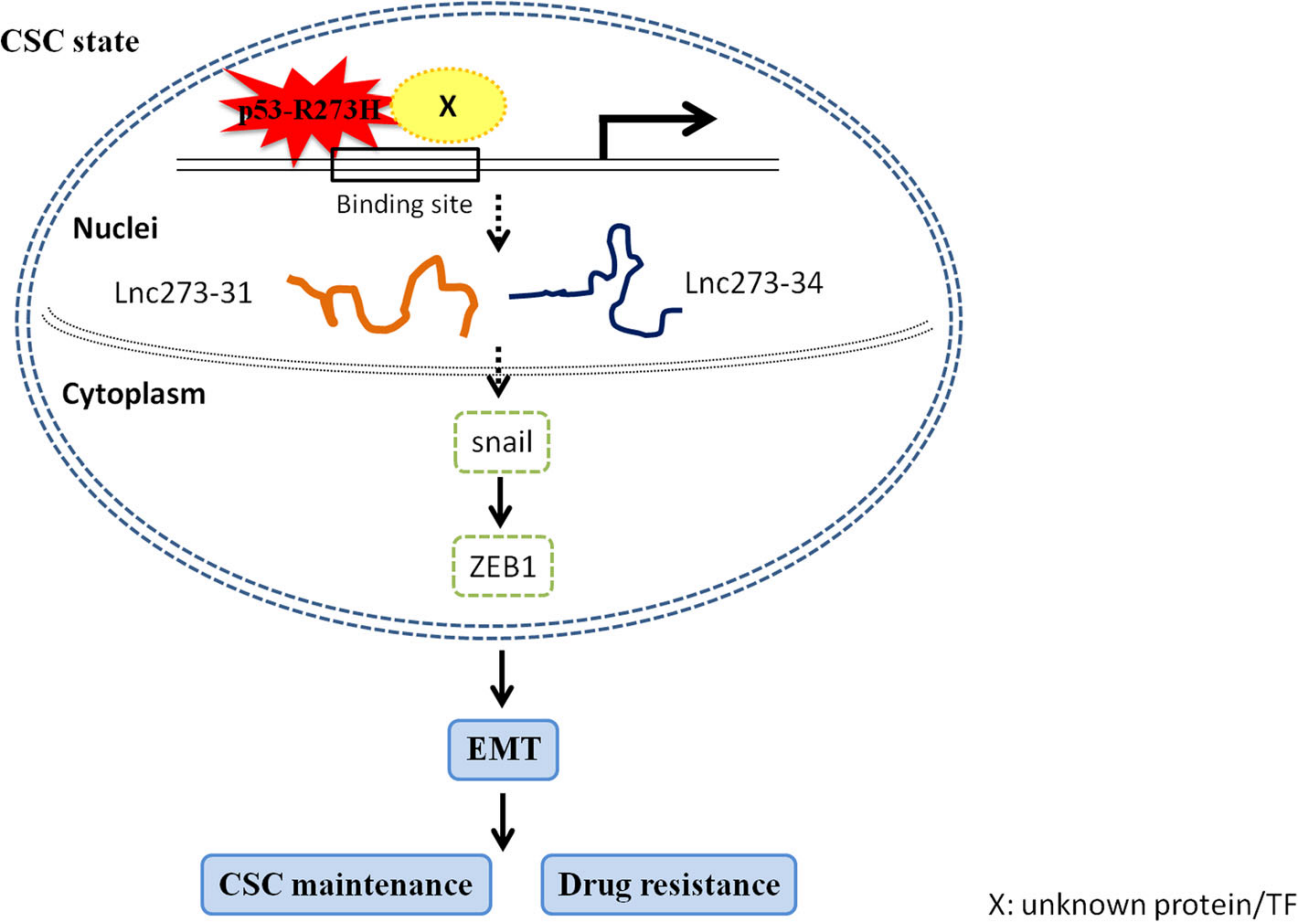
Schematic illustration of the possible mechanism by which lnc273–31 and lnc273–34, regulated by p53-R273H, promotes CSC self-renewal expansion and chemoresistance in colorectal cancer

Mutant p53 gained of new function mainly dependent on its stabilization and accumulation, as well as altering the functions of proteins or transcript factors that partnering with in the nuclei [43, 44]. In our study, lnc273–31 and lnc273–34 were identified combined RNA-seq with ChIP-seq, indicating the directly or indirectly binding with p53-R273H mutant. Also we proved that both lnc273–31 and lnc273–34 were mostly localized in the nuclei (Additional file 1: Figure S6C), which further supported the clue. Mutant p53 exhibited an oncogenic function through EMT progress had been reported many times [45]. It was proposed that EMT was able to acquire the ability to self-renewal by inducing a CSC characteristic [46, 47]. Many researchers have found that cancer cells showing EMT characteristics are resistant to chemotherapy, and cancer cells resistant to drugs exhibit EMT properties [48, 49]. Although EMT has been associated with stemness properties, in our study, the expression of key factors of EMT was not significantly increased assessed by Western blot (data not shown), which is consistent with another assumption, indicating EMT and stemness are not necessarily linked [50]. However, the expression of ZEB1 and snail, key factors of EMT progress, was significantly decreased when lnc273–31 or lnc273–34 knocking down, especially in p53-R273H spheroid cells (Additional file 1: Figure S7B), indicating the core regulatory function of lnc273–31 and lnc273–34 in EMT. The ZEB1 and snail regulatory axis in CSCs has been explored in plenty of studies [51–55]. Thus, we proposed the possible mechanism by which lnc273–31 and lnc273–34 regulated by p53-R273H enhancing colorectal cancer stem cell stemness and chemoresistance (Fig. 8). Although these findings are insufficient, they all suggest an important role for lnc273–31 and lnc273–34 in CSC maintenance and highlight the need for further investigation of the causal role of them in CSCs and chemoresistance and the underlying molecular mechanisms.

Recently, Ma et al. found that low expression of SH3PXD2A-AS1 (lnc273–34) is associated with colorectal cancer cell proliferation, migration and invasion in vitro as well as tumorigenesis inhibition in vivo, which validates the present sequencing results [56]. It is worth noting that the cell lines used in this article is SW480 and DLD1, which obtains the R273H and S241F mutation, respectively. S241F mutation is not as frequently mutated as R273H. However, both the R273H and the S241F mutation could affect DNA-binding activity, leading to dysregulation of p53 downstream target genes [57], suggesting another possible mechanism by which lnc273–31 and lnc273–34 accelerate colorectal cancer progression, which also needs further investigation.

LncRNAs play dynamic roles in chemoresistance [58] either with an intrinsic or an acquired chemoresistant phenotype through various mechanisms [59]. Although the chemoresistant mechanism was not specific clearly defined, the present results declared that a subset of lncRNAs involved in colorectal cancer stemness maintenance and to be essential for resistance to oxaliplatin. Thus, it is important to obtain a better understanding of the multiple components are involved in chemoresistance. Meanwhile, the present findings suggested that p53-R273H-regulated lncRNAs may be novel indicators or predictors for the prognosis of patients with p53-R273H, especially patients with metastasis, and may be used to overcome drug resistance in colorectal cancer therapy.

## Conclusions

In summary, the present study identified a set of lncRNAs regulated by p53-R273H, combined RNA-seq with ChIP-seq, required for colorectal cancer stem cell self-renewal, tumor propagation and chemoresistance. Further evidence suggested that lnc273–31 and lnc273–34 may serve as promising indicators or predictors for patients with p53-R273H mutation and are vital for chemotherapy.

## Additional files

Additional file 1: **Figure S1A.** Western blot analysis of HCT116 p53−/− over-expression p53 point mutants. **Figure S2A.** Schematic diagram of constructing HCT116 endogenous p53 point mutant (PM) cells. **Figure S2B.** Sanger sequencing of p53-R273H point mutant (R273H PM) representative cells. **Figure S3.** Global profiling and identification of p53-R273H-regulated lncRNAs. **Figure S3A.** Different types of transcripts. **Figure S3B.** Different types of noncoding transcripts. **Figure S3C.** A two-dimensional heatmap of 1957 lncRNAs. **Figure S3D.** Principal components analysis (PCA) for three independent replicates. The principal component accounted of p53-R273H and p53-ctrl were 85.7 and 69.6% respectively. **Figure S4A.** Validation of differentially expressed 41 lncRNAs by RT-qPCR. **Figure S5.** Genome-wide analysis of p53-R273H-regulated protein coding genes in CSC state. **Figure S5A.** A two-dimensional heatmap of 307 mRNAs. **Figure S5B.** Principal components analysis (PCA) for three independent replicates. **Figure S5C.** Hierarchical clustering for three independent replicates. **Figure S5D.** Signaling pathway based on KEGG enrichment analysis of p53-R273H-regulated coding genes in CSC state. **Figure S5E.** GO biological processes enrichment analysis of p53-R273H-regulated coding genes in CSC state. **Figure S5F.** Regulatory network construction of TFs (dark blue), lncRNAs (red) and mRNAs (light green). The average degree of lncRNAs was 46.39, higher than 35.39, the average degree of protein coding genes. **Figure S6A.** ChIP-qPCR for validating of the binding of p53 and the promotor of lnc273–31 or lnc273–34. **Figure S6B.** The expression levels in ALDH positive and ALDH negative cells sorted by FACS. **Figure S6C.** Subcellular localization of lnc273–31 and lnc273–34 was analyzed by RT-qPCR upon biochemical fractionation of p53-R273H speroid cells. **Figure S7A.** Quantitative real-time PCR analyzed the expression of stemness-related genes in HCT116 p53 PM cells. **Figure S7B.** Western blot analysis of ZEB1 and snail in lnc273–31 or lnc273–34 depletion cells. **Figure S8.** The association of age (S8A), gender (S8B), smoking (S8C), alcohol abuse (S8D), family history (S8E), lymphatic vessel (S8F), TNM stage (S8G-I) and the expression levels of lncRNAs in 25 colorectal cancer patients with or without p53-R273H mutation. **Table S1.** Primers for qPT-PCR. **Table S2.** Purchased ASO pool sequences. **Table S3.** Primers for ChIP-qPCR. (DOCX 2058 kb)

Additional file 2: The list of differentially expressed lncRNAs. (XLSX 11 kb)

Additional file 3: The results of KEGG and GO analysis. (XLSX 18 kb)

Additional file 4: The list of differentially expressed protein coding genes (PCGs). (XLSX 128 kb)

Additional file 5: LncRNA annotation. (XLSX 107 kb)

Additional file 6: The list of lncRNAs analyzed by RNA-seq combined with ChIP-seq. (XLSX 29 kb)

Additional file 7: Clinical patient information. (XLSX 11 kb)

## Data Availability

The datasets used and/or analyzed during the current study are available from the corresponding author on reasonable request.

## Abbreviations

ChIP-seq: ChIP-sequencing
CRC: Colorectal cancer
CSC: Cancer stem cell
ELDA: Extreme limiting dilution analysis
GO: Gene Ontology
GOF: Gain of oncogenic functions
KEGG: Kyoto Encyclopedia of Genes and Genomes
lncRNA: long noncoding RNA
LOF: Loss of tumor-suppressive functions
NC: Negative control
RNA-seq: RNA-sequencing
