# Supplementary material for: P53-R273H mutation enhances colorectal cancer stemness through regulating specific lncRNAs
Source: J Exp Clin Cancer Res. 2019 Aug 28;38:379. doi: 10.1186/s13046-019-1375-9 (PMC6712617; doi:10.1186/s13046-019-1375-9)
Supplement: Supplementary file 1 — Figure S1A. Western blot analysis of HCT116 p53−/− over-expression p53 point mutants. Figure S2A. Schematic diagram of constructing HCT116 endogenous p53 point mutant (PM) cells. Figure S2B. Sanger sequencing of p53-R273H point mutant (R273H PM) representative cells. Figure S3. Global profiling and identification of p53-R273H-regulated lncRNAs. Figure S3A. Different types of transcripts. Figure S3B. Different types of noncoding transcripts. Figure S3C. A two-dimensional heatmap of 1957 lncRNAs. Figure S3D. Principal components analysis (PCA) for three independent replicates. The principal component accounted of p53-R273H and p53-ctrl were 85.7 and 69.6% respectively. Figure S4A. Validation of differentially expressed 41 lncRNAs by RT-qPCR. Figure S5. Genome-wide analysis of p53-R273H-regulated protein coding genes in CSC state. Figure S5A. A two-dimensional heatmap of 307 mRNAs. Figure S5B. Principal components analysis (PCA) for three independent replicates. Figure S5C. Hierarchical clustering for three independent replicates. Figure S5D. Signaling pathway based on KEGG enrichment analysis of p53-R273H-regulated coding genes in CSC state. Figure S5E. GO biological processes enrichment analysis of p53-R273H-regulated coding genes in CSC state. Figure S5F. Regulatory network construction of TFs (dark blue), lncRNAs (red) and mRNAs (light green). The average degree of lncRNAs was 46.39, higher than 35.39, the average degree of protein coding genes. Figure S6A. ChIP-qPCR for validating of the binding of p53 and the promotor of lnc273–31 or lnc273–34. Figure S6B. The expression levels in ALDH positive and ALDH negative cells sorted by FACS. Figure S6C. Subcellular localization of lnc273–31 and lnc273–34 was analyzed by RT-qPCR upon biochemical fractionation of p53-R273H speroid cells. Figure S7A. Quantitative real-time PCR analyzed the expression of stemness-related genes in HCT116 p53 PM cells. Figure S7B. Western blot analysis of ZEB1 and snail in lnc273–31 or lnc273– [file 13046_2019_1375_MOESM1_ESM.docx]

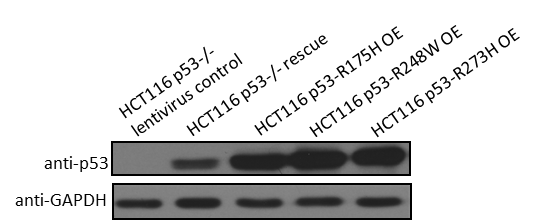


S1A. Western blot analysis of HCT116 p53-/- over-expression p53 point mutants.


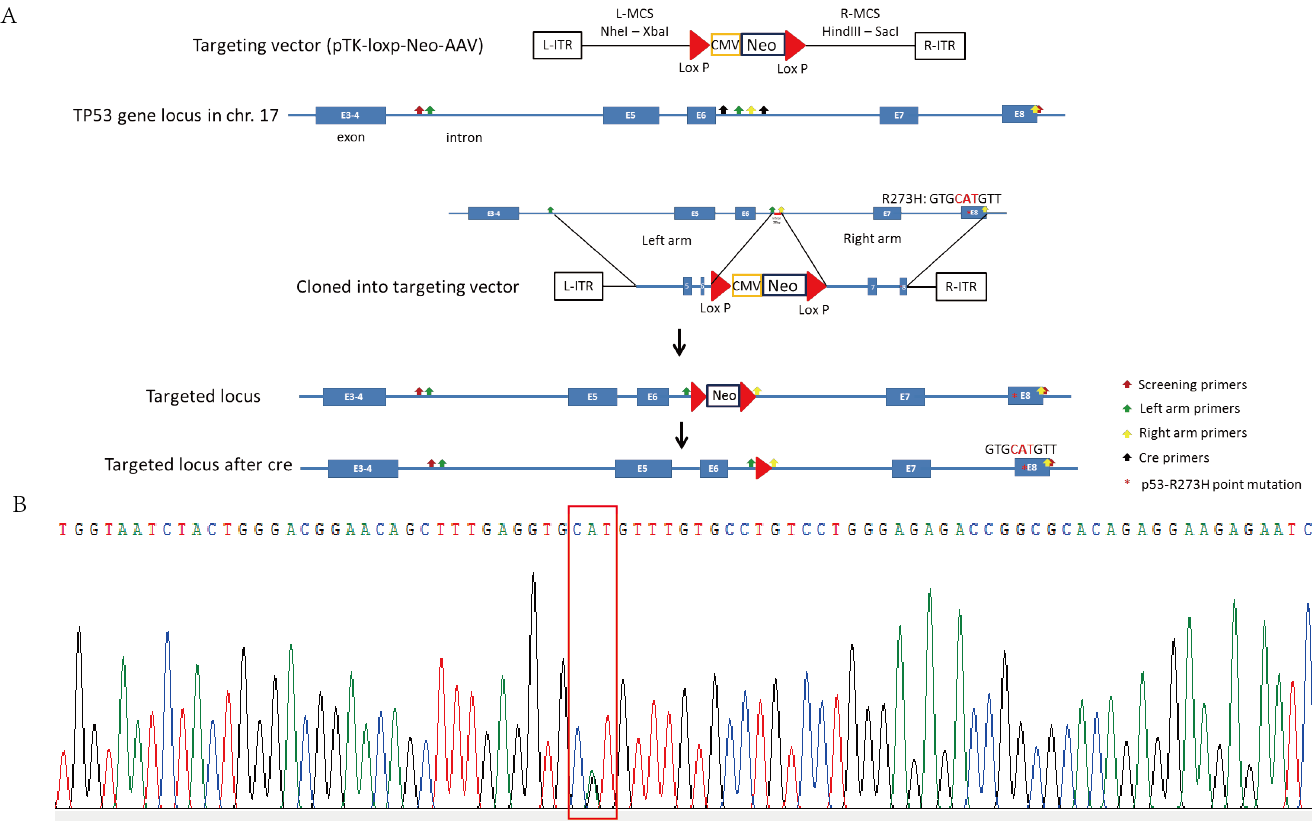


S2A. Schematic diagram of constructing HCT116 endogenous p53 point mutant (PM) cells.

S2B. Sanger sequencing of p53-R273H point mutant (R273H PM) representative cells.

Global profiling and identification of p53-R273H-regulated lncRNAs
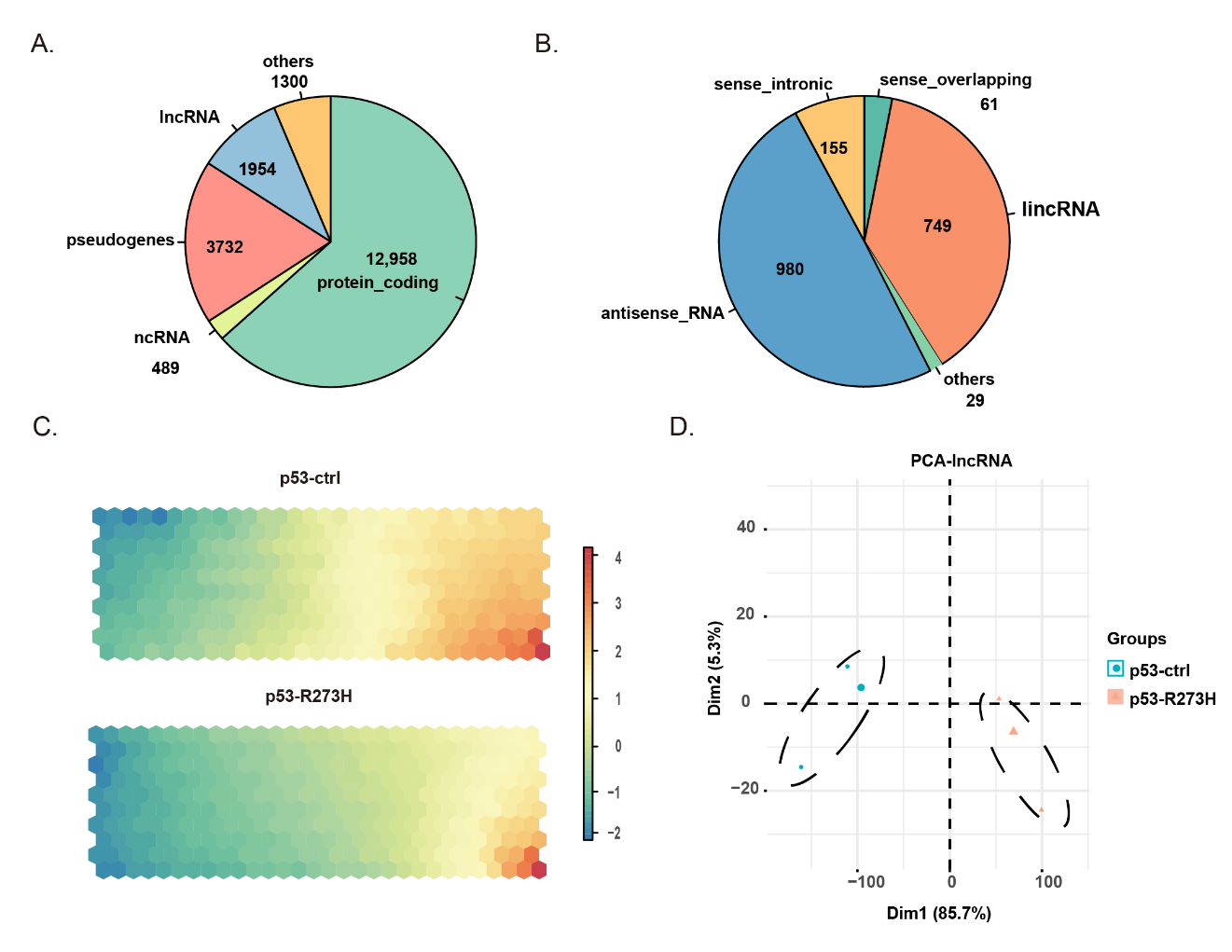


S3A Different types of transcripts.

S3B Different types of noncoding transcripts.

S3C A two-dimensional heatmap of 1,957 lncRNAs.

S3D Principal components analysis (PCA) for three independent replicates. The principal component accounted of p53-R273H and p53-ctrl were 85.7% and 69.6% respectively.







S4A. Validation of differentially expressed 41 lncRNAs by RT-qPCR.

A


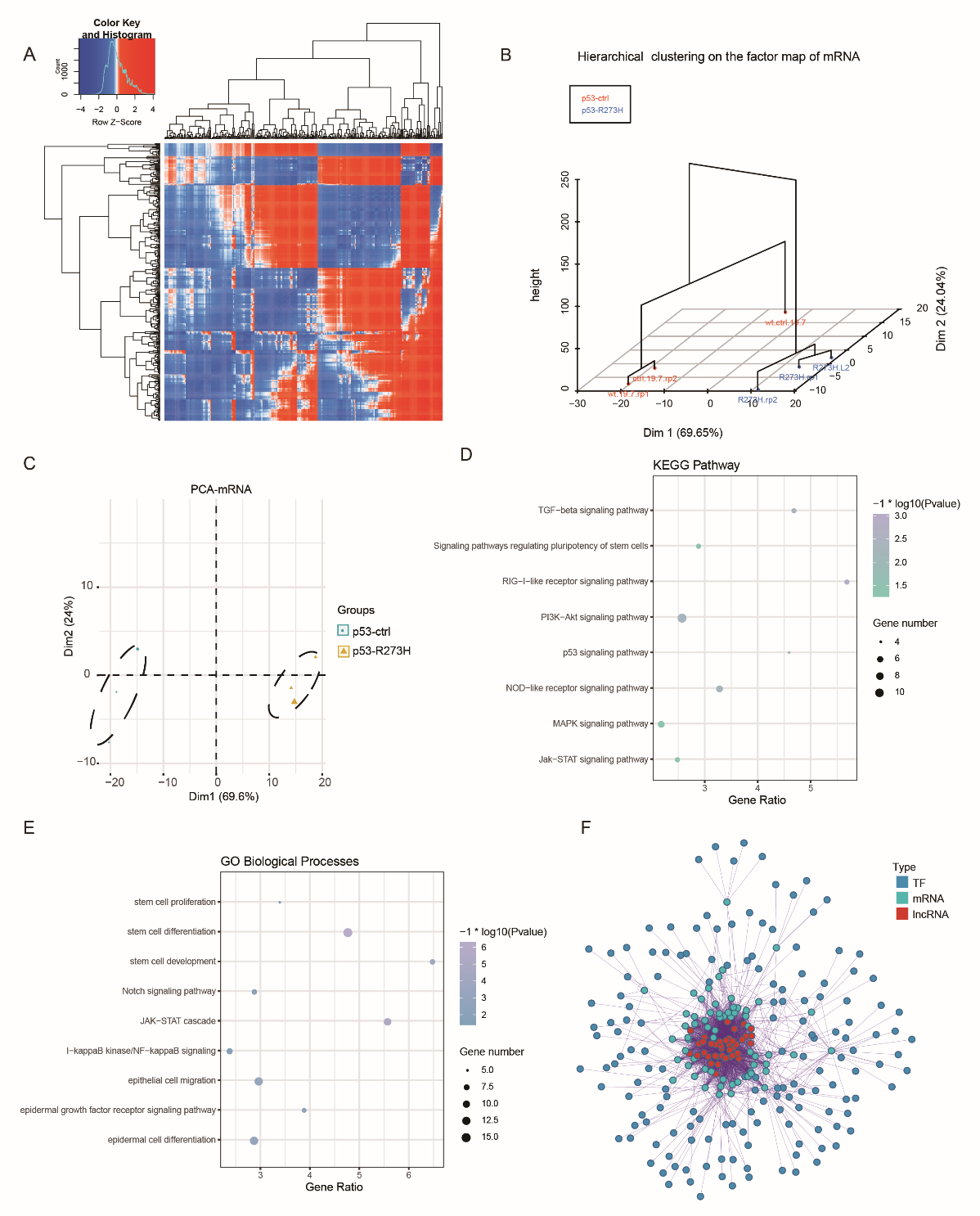
Genome-wide analysis of p53-R273H-regulated protein coding genes in CSC state.

S5A A two-dimensional heatmap of 307 mRNAs.

S5B Principal components analysis (PCA) for three independent replicates.

S5C Hierarchical clustering for three independent replicates.

S5D Signaling pathway based on KEGG enrichment analysis of p53-R273H-regulated coding genes in CSC state.

S5E GO biological processes enrichment analysis of p53-R273H-regulated coding genes in CSC state.

S5F Regulatory network construction of TFs (dark blue), lncRNAs (red) and mRNAs (light green). The average degree of lncRNAs was 46.39, higher than 35.39, the average degree of protein coding genes.


Fig S6A ChIP-qPCR for validating of the binding of p53 and the promotor of lnc273-31 or lnc273-34.



Fig S6B The expression levels in ALDH positive and ALDH negative cells sorted by FACS.

Fig S6C Subcellular localization of lnc273-31 and lnc273-34 was analyzed by RT-qPCR upon biochemical fractionation of p53-R273H speroid cells. Actin and DANCR^[[1]](#endnote-1)^ was used as controls for cytoplasmic transcripts, LncTCF7^[[2]](#endnote-2)^ and U2 were used as controls for nuclear transcripts.


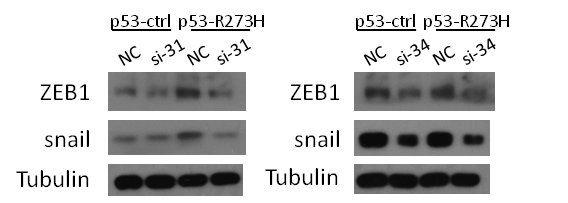
**
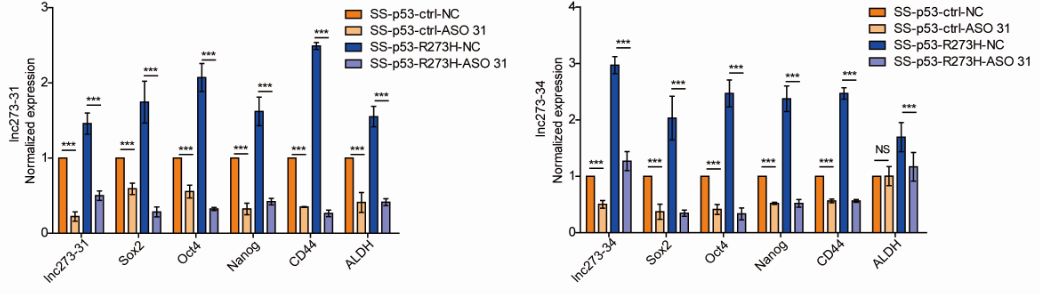
**

B

A

The gene expression changes when knocking down lnc273-31 or lnc273-34 in HCT116 p53 PM spheroid cells.

S7A. Quantitative real-time PCR analyzed the expression of stemness-related genes in HCT116 p53 PM cells.

S7B. Western blot analysis of ZEB1 and snail in lnc273-31 or lnc273-34 depletion cells.


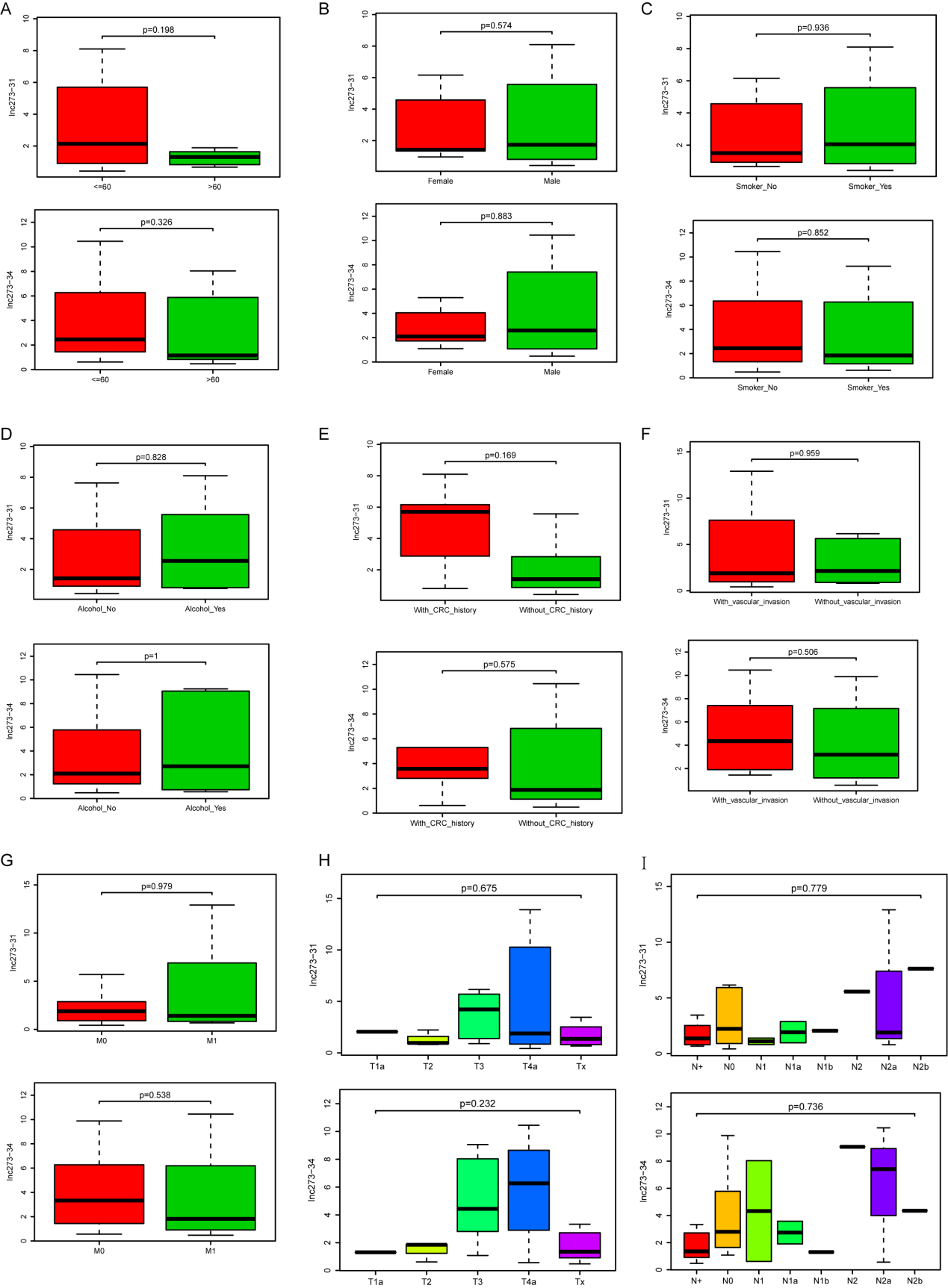


S8. The association of age (S8A), gender (S8B), smoking (S8C), alcohol abuse (S8D), family history (S8E), lymphatic vessel (S8F), TNM stage (S8G-I) and the expression levels of lncRNAs in 25 colorectal cancer patients with or without p53-R273H mutation.

**Methods**

**Antibodies**

Antibodies against p53 was purchased from abcam (ab1101). Antibodies against tubulin (T6199) was purchased from Sigma-Aldrich. Antibodies against the GAPDH (TA-08) was purchased from ZSGB-BIO. Antibodies against ZEB1 (3396) and snail (3879) were purchased from Cell Signaling Technology.

**Western blot**

Cells were washed with PBS, harvested, lysed with SDS lysis buffer (50 mM Tris–HCl [pH 6.8], 10% glycerol, and 2% SDS), and then quantified using BCA protein assay reagent (Pierce). The extracts were separated using 10% SDS-PAGE and were then electrophoretically transferred to a PVDF membrane (GE Healthcare) according to standard protocols. The membrane was blocked in 5% skim milk for 1 h at room temperature and then incubated overnight with the indicated antibodies at 4 °C.

**Isolation of RNA from nuclear and cytoplasm**

The separation of nuclear and cytoplasmic fractions was conducted using the Ambion® PARIS™ (Thermo Fisher, AM1921) as the manufacturer’s instruction. The nuclear and cytoplasmic RNA were then performed cDNA synthesis and RT-qPCR as described before.

**Construction of the regulatory network**

The regulation relationship between human TF and differentially expressed genes were acquired from TRRUST V2 [^[[3]](#endnote-3)^] database (www.grnpedia.org/trrust) as the background of interaction. Selected the interaction pairs between TF and differentially expressed genes according to the significance p value of hypergeometric test (less than 0.05).

A lncRNA–mRNA co-expression pair was defined for p53-R273H if it met all of the following criteria: corr (lncRNA, mRNA) < -0.4, P < 0.05 or corr (lncRNA, mRNA) >0.4, P < 0.05, where corr (lncRNA, mRNA) represent the Pearson correlation coefficient of lncRNA–mRNA based on established co-expression data. Regulatory network and TF/lncRNA/mRNA interactions were mapped using Cytoscape software [^[[4]](#endnote-4)^].

**SOM and PCA analysis**

To achieve a perspective outline for a dynamic expression pattern of lncRNAs and mRNAs, p53-R273H and p53-ctrl expression data were compiled and calibrated for establishing self-organizing maps (SOMs)^[^^[[5]](#endnote-5)]^ and Hierarchical clustering respectively. Based on the expression of 903 mRNAs and 1957 lncRNAs, two groups of samples were separated. To address the transcriptional role of p53-R273H on lncRNAs and mRNAs respectively, a principal components analysis (PCA)^[^^[[6]](#endnote-6)]^ was conducted. In total, the principal component accounted of p53-R273H and p53-ctrl were 85.7% and 69.6% respectively.

Table S1 Primers for qPT-PCR.

| Gene | Sequence |
| --- | --- |
| Lnc273-31-F | CTGATTACCTCCCACGTGCC |
| Lnc273-31-R | GGGCAAATTGCTTGCTGTCT |
| Lnc273-34-F | ACACACTTGCAAAAGCCCAC |
| Lnc273-34-R | TTCAGCCTGGCTCTGCTTAC |
| Actin-F | AAGGAGCCCCACGAGAAAAAT |
| Actin-R | ACCGAACTTGCATTGATTCCAG |
| Sox2-F | TGGACAGTTACGCGCACAT |
| Sox2-R | ACCTACAGCATGTCCTACTCG |
| Oct4-F | CTTGAATCCCGAATGGAAAGGG |
| Oct4-R | GAGGATCACCCTGGGATATACAC |
| Nanog-F | CCCCAGCCTTTACTCTTCCTA |
| Nanog-R | GACCTGGAACAATTCAACCTGG |
| RP11-288L9.4-F | GGAATCCGTTTCCTCTCCGC |
| RP11-288L9.4-R | GAATCCGCACAGAATCCGCA |
| CH17-80A12.1-F | GCAGGGAATTGTCACAGCAG |
| CH17-80A12.1-R | CCTGTGGATCCCGCACATTT |
| SERPINB9P1-F | GCTCTCAGCTGTTGGGGAAT |
| SERPINB9P1-R | TTATGGGGCTTGTGCGTTCT |
| TNRC6C-AS1-F | GGATTGGGGGAATGAACCCAG |
| TNRC6C-AS1-R | CAGATGCCAACTCTGTCGGG |
| RP11-817J15.2-F | TTTGAGCTCAGCCCTTCTCG |
| RP11-817J15.2-R | TGACCCTTGAGGGACAGGAA |
| RP11-89K21.1-F | AGCAAGACGCCCAGTTCATT |
| RP11-89K21.1-R | GGTGAGCTGGCGAATACTGA |
| BISPR-F | CTGTGCCTCTGCTGTGTGTTA |
| BISPR-R | GGAGACCGGACCAACAGTG |
| LINC01588-F | ATGGGTATGCGCTGCTTTTG |
| LINC01588-R | CACTTGCCTCCCCAGATAGC |

F: Forward primer; R: Reverse primer

Table S2 Purchased ASO pool sequences.

| Name | Sequence |
| --- | --- |
| Lnc273-31-ASO | CGGAATTCATGGGTACATA |
|  | TGTGCAAAGCCTCAGCATA |
|  | GCCATCCTTGTGCTCAGCA |
|  | ATAGGCTGCAATCAAGTTCA |
|  | AATAGTGATAGCATGAACCC |
|  | ACTGTGAACCTTACTGCAAC |
| Lnc273-34-ASO | CGACAGAGTGAAGCCTTCA |
|  | GGAAGAACGATCTCCCAAA |
|  | CTGAAGGGAACAGTATCTT |
|  | ACTTGCAAAAGCCCACTGGG |
|  | AGCCACGGCTTGCTTGCAGA |
|  | TTCGGAGGTTCCCATACCCA |

Table S3 Primers for ChIP-qPCR.

| Name | Sequence |
| --- | --- |
| 31-up-1F | ATTGTGGCTTTAGGTAGGCCC |
| 31-up-1R | CTGCCTCTCCATCACAGACT |
| 31-down-1F | GCCACACTCACAGTGGACA |
| 31-down-1R | GGGGCCGGATCTACAGAAAC |
| 34-up-1F | CCCATAGCGAAGGTCAGCAG |
| 34-up-1R | GAGGCAGCATCTTGGCTCTAA |
| 34-up-2F | GGCAGGAGTGAGGCCTAGAT |
| 34-up-2R | CTGACCTTCGCTATGGGGAT |

F:Forward primer; R: Reverse primer; up: up-stream; down: down-stream

**Supplemental reference**

1. Yuan SX, Wang J, Yang F, et al. Long noncoding RNA DANCR increases stemness features of hepatocellular carcinoma by derepression of CTNNB1. Hepatology. 2016 Feb;63(2):499-511. [↑](#endnote-ref-1)
2. Wang Y, He L, Du Y, et al. The long noncoding RNA lncTCF7 promotes self-renewal of human liver cancer stem cells through activation of Wnt signaling. Cell Stem Cell. 2015 Apr 2;16(4):413-25. [↑](#endnote-ref-2)
3. Han H, Cho JW, Lee S, et al. TRRUST v2: an expanded reference database of human and mouse transcriptional regulatory interactions. Nucleic Acids Res.46, 380-386 (2018). [↑](#endnote-ref-3)
4. Shannon P, Markiel A, Ozier O, et al. Cytoscape: a software environ¬ment for integrated models of biomolecular interaction networks. Genome Res.13, 2498–2504 (2003). [↑](#endnote-ref-4)
5. Kim DH, Marinov GK, Pepke S, Singer ZS, He P, Williams B, Schroth GP, Elowitz MB and Wold BJ. Single-cell transcriptome analysis reveals dynamic changes in lncRNA expression during reprogramming. Cell stem cell. 2015 Jan 8;16(1):88-101. [↑](#endnote-ref-5)
6. Jolliffe IT, Cadima J. Principal component analysis: a review and recent developments. Philos Trans A Math Phys Eng Sci. 2016 Apr 13;374(2065):20150202. [↑](#endnote-ref-6)
